# Dual Effect by Chemical Electron Transfer Enhanced siRNA Lipid Nanoparticles: Reactive Oxygen Species-Triggered Tumor Cell Killing Aggravated by Nrf2 Gene Silencing

**DOI:** 10.3390/pharmaceutics16060779

**Published:** 2024-06-07

**Authors:** Fengrong Zhang, Tobias Burghardt, Miriam Höhn, Ernst Wagner

**Affiliations:** 1 Pharmaceutical Biotechnology, Center for Nanoscience, Ludwig-Maximilians-Universität (LMU) Munich, 81377 Munich, Germany; tobias.burghardt@cup.uni-muenchen.de (T.B.); miriam.hoehn@cup.uni-muenchen.de (M.H.); 2 CNATM-Cluster for Nucleic Acid Therapeutics Munich, 81377 Munich, Germany; 3 Center for Nanoscience (CeNS), LMU Munich, 81377 Munich, Germany

**Keywords:** endosomal escape, chemical electron transfer, lipid nanoparticles, small interfering RNA, reactive oxygen species

## Abstract

Insufficient endosomal escape presents a major hurdle for successful nucleic acid therapy. Here, for the first time, a chemical electron transfer (CET) system was integrated into small interfering RNA (siRNA) lipid nanoparticles (LNPs). The CET acceptor can be chemically excited using the generated energy between the donor and hydrogen peroxide, which triggers the generation of reactive oxygen species (ROS), promoting endosomal lipid membrane destabilization. Tetra-oleoyl tri-lysino succinoyl tetraethylene pentamine was included as an ionizable lipopeptide with a U-shaped topology for effective siRNA encapsulation and pH-induced endosomal escape. LNPs loaded with siRNA and CET components demonstrated a more efficient endosomal escape, as evidenced by a galectin-8-mRuby reporter; ROS significantly augmented galectin-8 recruitment by at least threefold compared with the control groups, with a *p* value of 0.03. Moreover, CET-enhanced LNPs achieved a 24% improvement in apoptosis level by knocking down the tumor-protective gene nuclear factor erythroid 2-related factor 2, boosting the CET-mediated ROS cell killing.

## 1. Introduction

Lipid nanoparticles (LNPs), with their core component consisting of cationic or ionizable lipids, are employed to package negatively charged nucleic acids [1,2,3,4,5]. Ionizable lipids containing secondary and/or tertiary amines are advantageous due to chemical architecture flexibility, providing endosomal pH-specific responsiveness [6,7,8,9]. Specifically, their acidic-switchable structure provides protonation–deprotonation capabilities during the preparation process, collaborating with other constituents to shape amorphous cores [10,11,12]. Importantly, facilitating the nucleic acid transfer into the cytosol is another crucial mission of ionizable lipids, avoiding the fate of degradation in lysosomal/endosomal acidic environments [13,14,15].

Mechanistic studies aimed at elucidating the endosomal escape of ionizable LNPs have been proposed, mainly membrane fusion, as well as a phospholipid, flip-caused non-lamellar phase change and proton sponge effect [16,17,18,19,20]. The initial membrane destabilization in this process relies on endosomal acidification and involves a combination of positive-charged lipids with negatively charged host phospholipids [21]. Notably, pH-specific endosomal action avoids direct damage to the cytosolic membrane, thus minimizing cytotoxic side effects. Researchers have adopted numerous strategies to enhance nucleic acid cytoplasmic transfer [22,23,24,25]. Despite the clinical success of LNPs with small interfering RNA (siRNA) and messenger RNA (mRNA) transfer, the efficiency of endosomal escape remains suboptimal, with success rates observed at only 1–2% [26]. The application of higher therapeutic doses carries the risk of side effects [27,28,29].

One alternative option for improving endosomal escape is the incorporation of sensitizers into nanovehicles that respond to specific external triggers, such as photo/sono energy, adjustably facilitating the destabilization of the vehicle and endosomal target membranes, inducing cytoplasmic transfer [30,31,32,33,34,35,36,37,38]. For example, researchers reported on a porphyrin-LNP that enabled near-infrared light-induced siRNA endosomal release [38]. Such a photochemically triggered cytosolic delivery is suitable for superficial tissues that light can specifically address. Recently, we explored chemical electron transfer (CET)-assisted RNA interference (RNAi) as an alternative to photochemical internalization, which successfully generated reactive oxygen species (ROS) without external triggers [39]. Bis (2,4,6-trichlorophenyl) oxalate (TCPO) and gold nanoparticle were employed as CET donor and acceptor, respectively. With the assistance of a donor and H_2_O_2_, the acceptor is activated, generating singlet oxygen (^1^O_2_) to disrupt endosomes and release siRNA formulated as lipopolyplexes into cytosol.

**Scheme 1:**
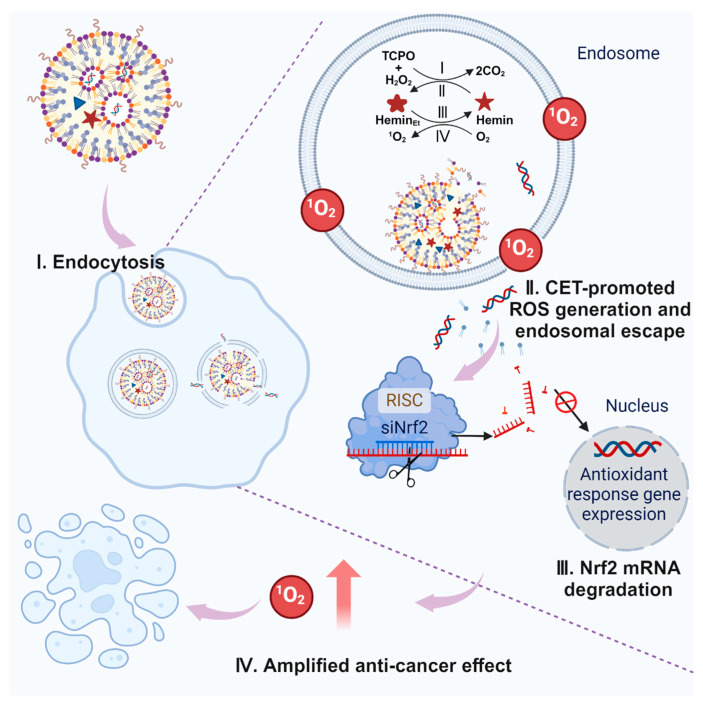
Illustrations of CET-induced siRNA endosomal escape and siNrf2-aggravated ROS cancer cell killing. The blue triangle inside the LNP denotes TCPO and red pentagram represents hemin.

Here, for the first time, we applied CET as an internal trigger for the enhanced cytosolic delivery of LNP loaded with siRNA (siRNA@Lipid) and enhanced ROS-triggered tumor cell killing. For this purpose, novel LNP compositions were packaged with siRNA and various doses of TCPO and hemin as CET donor and acceptor, respectively (Scheme 1). Given the excellent capacity of hemin and TCPO to induce ROS production in cancer cells, we sought to enhance gene silencing via endosomal membrane lipid peroxidation: the CET effect would induce the endosomal accumulation of ROS, accelerating the transfer of the gene sequence to the cytoplasm. Effective silencing of gene targets at low siRNA doses demonstrated an LNP-integrating CET-enhancing effect. Simultaneously, antitumoral siRNA extended the lifetime of ROS by silencing the oxidative stress protein, nuclear factor erythroid 2-related factor 2 (Nrf2), promoting cancer cell-killing efficiency.

## 2. Materials and Methods

More information about the methods is provided in the Supporting Information.

### 2.1. Materials and Chemicals

TCPO, hemin, 9,10-diphenanthraquinone (DPA), Roswell Park Memorial Institute (RPMI)-1640 medium, Dulbecco’s modified Eagle’s medium (DMEM), antibiotics, fetal bovine serum (FBS), MTT, 4′,6-diamidino-2-phenylindole (DAPI), paraformaldehyde (PFA), and rhodamine-phalloidin were sourced from Sigma-Aldrich (Munich, Germany). Cholesterol, distearoylphosphatidylcholine (DSPC), and 1,2-dimyristoyl-rac-glycero-3-methoxypolyethylene glycol-2000 (DMG-PEG) were purchased from Avanti Polar Lipids (Birmingham, AL, USA). 4-(2-hydroxyethyl)-1-piperazineethanesulfonic acid (HEPES) was bought from Biomol (Hamburg, Germany). GelRed^TM^ and peqGOLD Total RNA kits were obtained from VWR (Darmstadt, Germany). Quant-iTTM RiboGreen^®^ RNA assay kit and CellROX Green reagent were purchased from Invitrogen (Carlsbad, CA, USA). The luciferase assay kit was obtained from Promega (Madison, WI, USA). Fluorescein isothiocyanate (FITC)/propidium iodide (PI) apoptosis kit was obtained from R&D Systems (Minneapolis, MN, USA). qScript^TM^ cDNA SuperMix was purchased from Quantabio (Beverly, MA, USA). Reverse transcription-quantitative real-time PCR (RT-qPCR)-Mastermix was sourced from Thermo Fisher Scientific (Waltham, MA, USA). The ionizable lipopeptide tetra-oleoyl-tri-lysino-succinoyl tetraethylene pentamine (TOTL-Stp) with U-shape topology and the sequence (N- to C-terminus) K(OleA)_2_-Stp-K(OleA)-K(OleA)-OH was synthesized according to protocols described in previous publications [40,41,42], employing solid-phase assisted peptide chemistry with 2-chlorotrityl chloride resin as the solid support.

### 2.2. Preparation of LNPs

LNPs were formulated using a rapid-mixing method. First, cholesterol (5 mg mL^−1^), DSPC (2.5 mg mL^−1^), PEG-DMG (1 mg mL^−1^), and ionizable lipopeptide (10 mg mL^−1^) stock solutions were prepared in ethanol. The amounts of ionizable lipopeptide were calculated according to siRNA dose, hemin dose, and N/P or N/(P+C) ratios, where N is the molar quantity of ionizable nitrogen, P represents the molar quantity of phosphate group, and C corresponds to the molar quantity of carboxyl group derived from the anionic hemin content. The amounts of other lipids were determined using the molar ratio (Table S1). The lipids ethanol solutions were mixed, and the mixture was then combined with a dimethyl sulfoxide solution (*v*/*v*, 1/1) containing TCPO (9 mg mL^−1^) and hemin (1 mg mL^−1^). The total volume was 30 µL. After that, a siRNA aqueous solution (dissolved in sodium citrate buffer, 10 mM, pH 4) was rapidly mixed with the organic solution at a volume ratio of 3:1 and incubated at room temperature (RT) for 20 min. The resultant LNP solution was dialyzed in HEPES buffer (20 mM, pH 7.4) at 4 °C for 4 h to remove organic solvents. The final volume was 200 µL.

### 2.3. DPA Degradation Assay

A mixture was prepared by mixing 12 µL of a 1 mg mL^−1^ DPA solution with 25 µL of a hemin solution (25 µg mL^−1^), 15 µL of a TCPO solution (9 mg mL^−1^), 300 µL of H_2_O_2_ with various concentrations (1.56, 3.13, 6.25, 13.3, and 26.6 mM), and 448 µL of H_2_O. Subsequently, the mixture was measured using a UV–vis spectrophotometer (Agilent Technologies, Santa Clara, CA, USA) at different incubation times (2 min, 30 min, 1 h, and 2 h).

For the LNP-related ROS generation test, a reaction solution was similarly prepared by mixing 12 µL of a 1 mg mL^−1^ DPA solution with 25 µL of a Hemin+TCPO+siRNA@Lipid HEPES buffer, 300 µL of H_2_O_2_ (13.3 mM), and 463 µL of H_2_O. For Hemin+TCPO+siRNA@Lipid, different TCPO/hemin molar ratios (20, 40, 60, 80, 100, 150, and 200) were prepared. The N/P ratio was 3, the siRNA amount was 1 μg, and the TCPO amount was 80 μg. The molar ratio of different lipids was 50/38.5/10/1.5 (ionizable lipopeptide/cholesterol/phospholipid/DMG-PEG). Finally, the absorption of the solution was analyzed after incubation for 2 min and 2 h, respectively.

### 2.4. Gene Silencing Efficiency Assay

KB/eGFPLuc cells, N2a/eGFPLuc cells, and DU145/eGFPLuc [40,43] were utilized to optimize the LNP formulations with different N/P or N/(P+C) ratios. siGFP was used to silence eGFPLuc; the siCtrl was chosen to assess non-specific transfection effects. Cells were seeded in 96-well plates at a density of 5000 cells/well in a standard cell culture medium containing 10% FBS one day before the treatments. Afterward, the medium was replaced with 80 µL of fresh medium (containing 10% fetal bovine serum, 100 U mL^−1^ penicillin, and 100 μg mL^−1^ streptomycin) and 20 µL of LNP solution. The final amounts of siRNA were 100, 50, 25, 12.5, 6.25, 3.125, and 1.625 ng. The plates were incubated at 37 °C for 2 days. Then, the medium was removed, and 100 µL of cell lysis solution was added to fully lyse the cells by incubating at RT for 30 min. Following that, the luciferase activity was measured using a Centro LB 96 plate reader luminometer (Berthold, Bad Wildbad, Germany) with the help of a luciferin-LAR buffer solution. The transfection efficiency was calculated as a percentage of luciferase gene expression relative to HEPES buffer-treated control cells.

### 2.5. Cellular Uptake Study

KB cells were plated in 96-well plates at a density of 10,000 cells/well one day prior to the treatments. After that, the medium was replaced with 80 µL fresh DMEM medium (containing 10% fetal bovine serum, 100 U mL^−1^ penicillin, and 100 μg mL^−1^ streptomycin) and 20 µL of carrier solution. The nanocarrier solution contained 100 ng siCy5 and 4 μg hemin with an N/(P+C) ratio of 9. Following incubating the cells for different times (45 min, 4 h, 8 h, 12 h, and 24 h), 100 µL of PBS containing 500 IU heparin was introduced to clear away particles adhering to the cell. During the process, cells were incubated on ice for 30 min. Finally, the cells were collected, suspended in PBS with 10% FBS, and measured by flow cytometer (Beckman Coulter, Fullerton, CA, USA) in Cy5 channel (Ex: 635 nm, Em: 665 nm).

### 2.6. Endosomal Escape Assay

The endosomal escape of the CET system was studied using confocal laser scanning microscopy (CLSM, Leica, Wetzlar, Germany) images with the HeLa-galectin-8 (Gal8)-mRuby3 cell line, stably expressing mRuby3-Gal8 fusion protein [44,45]. HeLa-Gal8-mRuby3 cells were seeded in an 8-well Lab-Tek chamber at a density of 10,000 cells/well. The following day, fresh medium containing 10% fetal bovine serum, 100 U mL^−1^ penicillin, 100 μg mL^−1^ streptomycin, and LNPs was added, followed by incubation of the cells for 4 h, 12 h, 24 h, or 48 h. Subsequently, the cells were washed with PBS and fixed in a 4% PFA solution for 45 min at RT. DAPI and rhodamine-phalloidin were used to stain the cell nuclei and actin, respectively. Lastly, the cells were treated with PBS again to remove excess dye before observation with a CLSM.

### 2.7. Cell Apoptosis Assay

Twelve-well plates were prepared with KB cells seeded at a density of 20,000 cells/well 24 h prior to the treatments. Next, cells were incubated with different formulations for 48 h. The siRNA amount was 1.88 µg well^−1^, the TCPO amount was 60 µg well^−1^, and the hemin amount was 975 ng well^−1^. Then, cells were collected and washed with PBS to remove LNPs. The cells were suspended in 100 µL Annexin V binding buffer containing 1 µL Annexin V-FITC and 10 µL PI and incubated at RT for 15 min. Subsequently, the collected cells were measured by the flow cytometer.

### 2.8. RT-qPCR Assay

One day before treatment, KB cells were seeded in 6-well plates at a density of 120,000 cells/well. Subsequently, the medium was replaced with 1600 µL of fresh medium (containing 10% fetal bovine serum, 100 U mL^−1^ penicillin, and 100 μg mL^−1^ streptomycin) and 400 µL of HEPES buffer (20 mM, pH 7.4) containing LNPs. Each well contained 400 µg of TCPO, 6.5 µg of hemin, and 2 µg of siRNA. After incubation for 48 h, KB cells were collected from the different treatment groups, and total RNA was extracted using the peqGOLD Total RNA kit. cDNA was synthesized using qScript^TM^ cDNA SuperMix. Subsequently, RT-qPCR was conducted on a LightCycler 480 system using UPL Probes (Roche, Mannheim, Germany), TaqMan^®^ Gene Expression, and Mastermix Assays. The comparative cycle threshold (Ct) method was employed to calculate the relative abundance of Nrf2 mRNA and EG5 mRNA, with GAPDH mRNA serving as the housekeeping gene. Every assay was performed in triplicate.

### 2.9. Statistical Analysis

Data were expressed as the means ± standard deviation of at least three independent experiments. The statistical significance of the experiments was determined using the two-tailed Student’s *t*-test (*** *p* ≤ 0.001, ** *p* ≤ 0.01, * *p* ≤ 0.05).

## 3. Results and Discussion

### 3.1. Design and Preparation of LNPs

**Figure 1:**
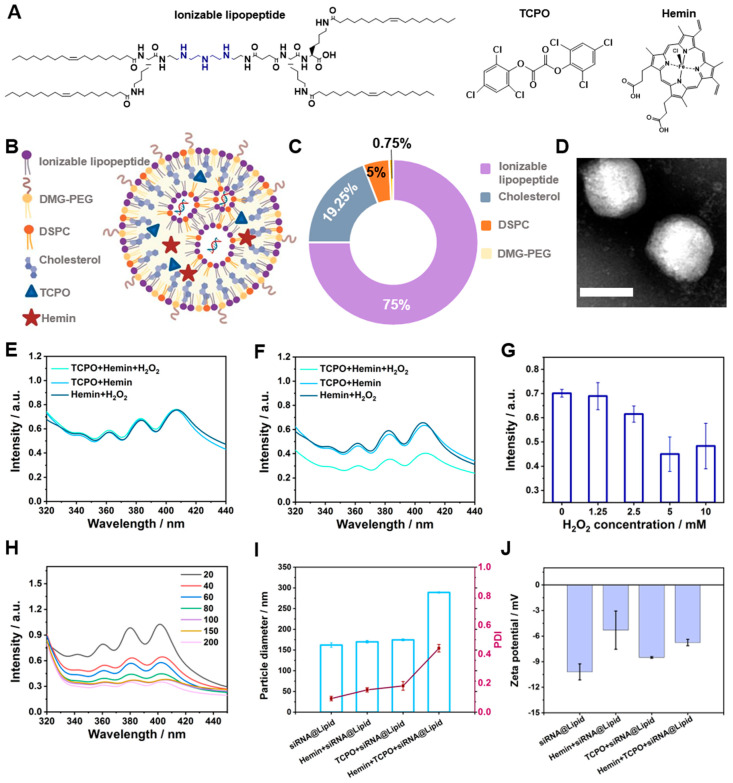
Design and preparation of LNPs. (**A**) Chemical structures of the ionizable lipopeptide TOTL-Stp, TCPO, and hemin used in the LNP formulation. (**B**) Schematic representation of the compounds in the CET-based LNP. (**C**) The molar ratio of each compound in the top-performing LNP. (**D**) The TEM image of LNPs contained hemin and TCPO in 20 mM HEPES buffer (pH 7.4). The N/(P+C) ratio was 9, the siRNA amount was 1 µg, the hemin amount was 1.3 µg, and the TCPO amount was 80 µg. The molar ratio of different lipids was 75/19.25/5/0.75 (ionizable lipopeptide/cholesterol/phospholipid/DMG-PEG). The scale bar is 100 nm. ROS-based degradation of DPA spectra under different conditions. (**E**) T = 2 min and (**F**) T = 2 h. (**G**) Ultraviolet–visible absorption intensity of DPA for LNPs at 383 nm under varying concentrations of H_2_O_2_. Reaction time was 2 h. The DPA, TCPO, and hemin concentrations used in the DPA degradation assay were 15 μg mL^−1^, 0.17 mg mL^−1^, and 0.78 μg mL^−1^, respectively. (**H**) Degradation spectra of DPA after adding different LNPs with a range of TCPO/hemin molar ratios. The concentration of H_2_O_2_ used was 5 mM. T = 2 h. The N/(P+C) ratio was 3, the siRNA amount was 1 μg, and the TCPO amount was 80 µg. The molar ratio of different lipids was 50/38.5/10/1.5 (ionizable lipopeptide/cholesterol/phospholipid/DMG-PEG). (**I**) Size distributions, PDI, and (**J**) zeta potentials of different formulations. Formulations containing 1 μg of siRNA in a total volume of 200 μL were diluted 1:5 with HEPES buffer before measurement.

Figure 1A–C presents the design of the novel LNPs. The ionizable lipopeptide TOTL-Stp provides siRNA binding and endosomal escape capabilities through its three cationizable secondary amines (Figure 1A). TOTL-Stp was synthesized by solid-phase supported peptide synthesis, as previously reported (Figure S1, Supporting Information) [40,41,42]. The four oleic acid tails were incorporated in a U-shaped sequence, K(OleA)_2_-Stp-K(OleA)-K(OleA)-OH, via bridging lysines, and they promote LNP stabilization through hydrophobic interactions and provide a fusogenic ability with the endosomal host membrane. LNPs were prepared using the standard solvent-exchange deposition method. In detail, helper lipids (cholesterol and phospholipid), poly(ethylene glycol) (PEG) lipid, ionizable lipopeptide, TCPO, and hemin were dissolved in an ethanol and dimethyl sulfoxide-containing organic solvent, and siRNA was dispersed in acidic sodium citrate buffer. The TCPO and hemin were encapsulated in the hydrophobic interlayer by rapidly adding the aqueous solution containing siRNA (Figure 1B and Figure S2, Supporting Information). Both electrostatic and hydrophobic interactions guided the assembly process. The top-performing formulation was derived from the LNP formulation of Onpattro^®^, increasing the ionizable lipopeptide content in accordance with the nitrogen/phosphate (N/P) ratio while maintaining the other components constant. The best formulation had a N/P or N/(P+C) ratio of 9 and a molar ratio of 75/19.25/5/0.75 (ionizable lipopeptide/cholesterol/phospholipid/DMG-PEG) (Figure 1C). Specifically, N represents the molar amount of ionizable nitrogen, P denotes the molar amount of the phosphate group, and C corresponds to the molar amount of the carboxyl group derived from the anionic hemin content. Transmission electron microscopy (TEM) revealed the morphological characteristics of the nanoparticles loaded with hemin and TCPO, exhibiting a relatively regular and spherical shape (Figure 1D).

ROS generation is the prerequisite for the CET effect-enhanced endosomal escape of siRNA. In the current light-free CET system, the lipophilic oxalate derivative TCPO and the acceptor hemin co-localized in the hydrophobic section, ensuring an energy transfer distance of less than 10 nm. As displayed in Figure S3 (Supporting Information), the reaction between TCPO and H_2_O_2_ generates an energy-rich dioxetanedione. The intermediate excites hemin from the ground singlet state to the excited singlet state through energy transfer rather than light energy. Subsequently, through intersystem crossing, an excited triplet state is formed, that further reacts with oxygen to produce ^1^O_2_. To analyze ^1^O_2_, DPA, characterized by three distinctive absorption peaks, was adopted. Notably, in the CET group, the signal of DPA significantly decreased to 72% after 1 h and further dropped to 59% within 2 h. In comparison, without the assistance of H_2_O_2_ or TCPO, the retention rate was still higher than 90% after 2 h (Figure 1E,F and Figure S4, Supporting Information). Furthermore, H_2_O_2_-dependent DPA oxidation was further evidenced by elevating H_2_O_2_ concentration from 0 to 5 mM (Figure 1G). These findings strongly evidenced the excellent ability of CET-based light-free systems to induce ROS production through energy transfer.

Building on the foundation of the CET effect, we expanded the system by integrating siRNA, cholesterol, and lipids, thereby creating the CET-based LNPs (Hemin+TCPO+siRNA@Lipid). Since ROS generation can vary with the molar ratio of TCPO/hemin, we optimized the DPA signal of various formulations using different molar ratios (Figure 1H). The average hydrodynamic sizes of the formulations measured using dynamic light scattering (DLS) exhibited minimal variation (Figure S5A, Supporting Information, nanoparticle diameters in blue). Importantly, the monodispersity of all formulations was well proved by low polydispersity index (PDI) values, which were all around 0.2 (Figure S5A, Supporting Information, PDI data in red). The optimal TCPO/hemin molar ratio, which was determined to be 80, was utilized in the following experiments (Figure S5B,C, Supporting Information). In addition, DLS showed that the ionizable lipid composition and siRNA formed LNPs, with or without TCPO and hemin, holding hydrodynamic sizes ranging from 162 nm and 289 nm and ζ-potentials among –5 mV and –10 mV (Figure 1I,J). In the case of the CET reaction, it was observed that the UV-vis absorption of TCPO at 400 nm disappeared by reacting with H_2_O_2_, which can be attributed to the decomposition of TCPO (Figure S6, Supporting Information).

### 3.2. Optimization of LNPs

**Figure 2:**
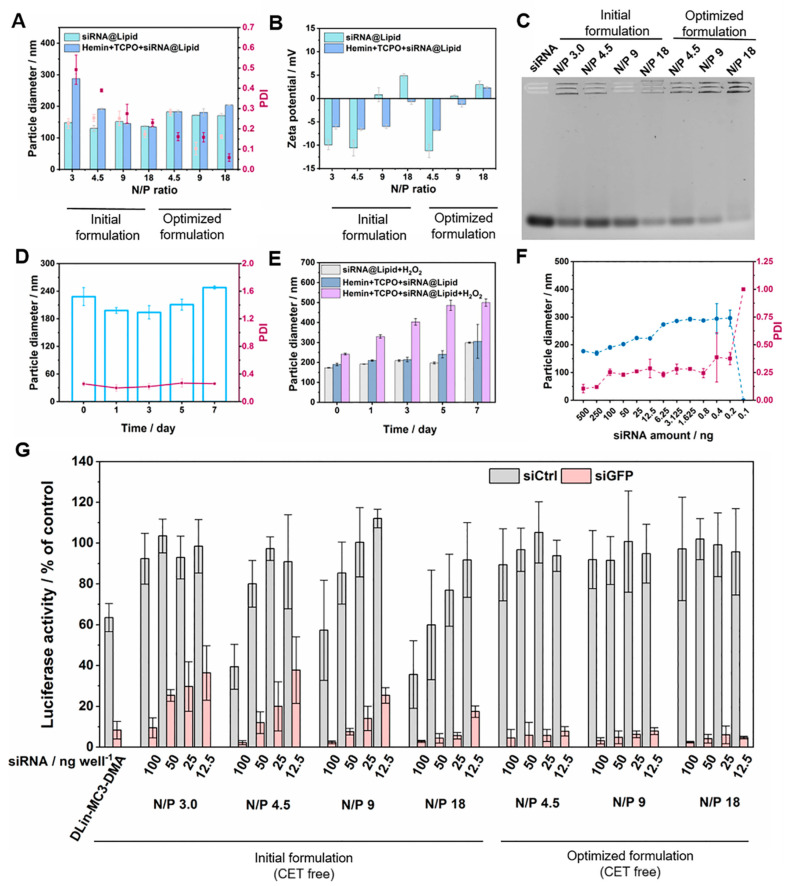
Optimization of LNPs. (**A**) Size distributions, PDI, (**B**) zeta potentials, and (**C**) siRNA binding assay of initial and optimized formulations at different N/P ratios. The siRNA@Lipid group was formulated without hemin and TCPO additions. All formulations for the siRNA binding assay were CET-enhanced LNPs (Hemin+TCPO+siRNA@Lipid). The gel electrophoresis conditions were 2.5% agarose gel and 100 V, and the running time was 60 min. For size, PDI, and zeta potential measurements, the siRNA amount was 1 μg, the hemin amount was 1.3 µg, and the TCPO amount was 80 µg. For the gel shift assay, the siRNA amount was 100 ng, the hemin amount was 0.13 µg, and the TCPO amount was 8 µg. (**D**) Stability assay of CET-enhanced LNPs after storing at 4 °C for one week. The blue bars indicate particle diameter and the red line denotes the PDI value. (**E**) Effect of CET reaction and (**F**) dose dilution on the CET-enhanced LNPs’ size (blue line) and PDI (red line) measured using DLS. The test conditions were the same as (**A**). (**G**) Gene silencing efficiency of KB/eGFPLuc cells treated with various CET-free LNPs (siRNA@Lipid) at siRNA doses of 12.5, 25, 50, and 100 ng for 48 h. DLin-MC3-DMA was formulated as LNPs at an N/P ratio of 3 and a siRNA dose of 500 ng well^−1^. Luciferase activity was normalized to cells without any treatment.

An elevated N/P ratio in LNP formulations may contribute to higher gene silencing efficacy but also amplify potential toxicity issues. To address these conflicting requirements, we optimized the formulation by selectively improving the ionizable lipid content according to the N/P ratio and keeping the other components constant. As indicated in Table S1, the optimized LNPs possessed ionizable lipopeptide ratios ranging from 60 to 86%, with the corresponding ratio of PEG lipid reduced from 1.2 to 0.4%, significantly lower than that of the Onpattro^®^ formulation (1.5%). In general, all LNPs exhibited a favorable size range of 130–183 nm and a PDI range of 0.10–0.28 without hemin and TCPO (Figure 2A). Adjusting the N/P ratio had a negligible impact on the hydrodynamic size and dispersion of optimized LNPs. The ζ-potentials of optimized LNPs shifted from negative to positive at N/P 9 (Figure 2B). In contrast, after incorporating CET components, the size of siRNA@Lipid at N/P 3 increased to 288 nm, and low dispersibility in the HEPES buffer was observed. Notably, for the optimized formulations, encapsulating hemin and TCPO led to a slight increase in size, and their monodispersity was maintained. The loading efficiency of the TCPO and hemin were calculated. Specifically, the formulation with N/P 9 was the top performer, displaying encapsulation efficiencies of >60% for hemin and >20% for TCPO (Figure S7, Supporting Information).

To investigate the siRNA binding ability of CET-enhanced LNPs, a gel shift assay was performed using a 2.5% agarose gel (Figure 2C). The binding was significantly potentiated when the fraction of ionizable lipopeptide was increased in LNPs. The results indicated the specific elevation in ionizable lipopeptide content substantially enhanced siRNA binding efficiency. Particularly, when the N/P ratio of the optimized formulation reached or exceeded 9, superior siRNA binding capabilities were observed. In addition, the storage stability of the new formulation was evaluated. The formulations showed no considerable change in size over one week at 4 °C (Figure 2D). Oppositely, a substantial increase in size and PDI was recognized upon introducing the H_2_O_2_, and this phenomenon was further amplified with prolonged incubation at 37 °C (Figure 2E and Figure S8, Supporting Information). The CET-dependent size alteration of LNPs likely corresponds to CO_2_ generation within the core and rearrangement events that might be induced by lipid peroxidation. Previous research has demonstrated that the double bonds in phospholipids can be oxidized in the presence of ^1^O_2_ [46,47]. Next, we tested the influence of dilution on integrity. The nanoparticles were prepared with a high siRNA amount of 500 ng, followed by serial dilution to lower concentrations. Encouragingly, the formulations exhibited excellent dilution stability at relatively low doses (Figure 2F). It has been reported that the hydrophilicity of PEG-lipid contributes to the steric stabilization of LNP, ensuring excellent stability even under high ionic strength conditions. So, the RNA leakage assay of CET-enhanced LNPs was investigated following exposure to heparin and sodium chloride at series concentrations (Figure S9, Supporting Information). At a relatively high sodium chloride concentration (1.25 M), LNPs exhibited the robust encapsulation of siRNA. With increased ionic strength, slightly stronger Ribogreen fluorescence was observed, indicating that some siRNA was released from dissociated LNPs. This observation is consistent with the trends in the heparin competition assay.

Before we explored the CET-enhanced gene silencing, the efficiency of CET-free LNPs was first analyzed after incubation with serial concentrations of LNPs for 48 h (Figure 2G). The transfections were carried out in KB/eGFPLuc cells, which express eGFP-luciferase (eGFPLuc) fusion protein. Target gene silencing was assessed by measuring luciferase activity. The MC3 LNP formulation dosed at 500 ng siRNA/well was selected as a positive control. Both the initial and the optimized CET-free LNPs effectively silenced eGFPLuc gene expression at siGFP concentrations that varied from 12.5 to 100 ng per well. Conversely, the siCtrl groups of the corresponding initial formulations exhibited noticeable intrinsic cytotoxicity. Specifically, in the range of N/P ratios from 4.5 to 18, the luciferase activity gradually decreased with the escalating dose of siCtrl in LNPs. Encouragingly, all optimized LNPs loaded with siCtrl showed nearly no reduction in luciferase activity; meanwhile, the excellent gene silencing capability was validated, with all formulations containing siGFP at 50 ng mediating more than 90% gene silencing efficiency. By comparison, the MC3 LNPs exhibited the same efficiency, with a 10-fold higher siRNA amount. Similar conclusions were also demonstrated in DU145/eGFPLuc and N2a/eGFPLuc cells (Figure S10, Supporting Information). Considering both the CET effect and RNAi efficacy, LNP with N/P 9 was identified as the top performer and utilized in the subsequent experiments. Its siRNA encapsulation efficiency was determined to be 98% using the Ribogreen assay.

### 3.3. Evaluation of Cellular Uptake and Cellular ROS Production

**Figure 3:**
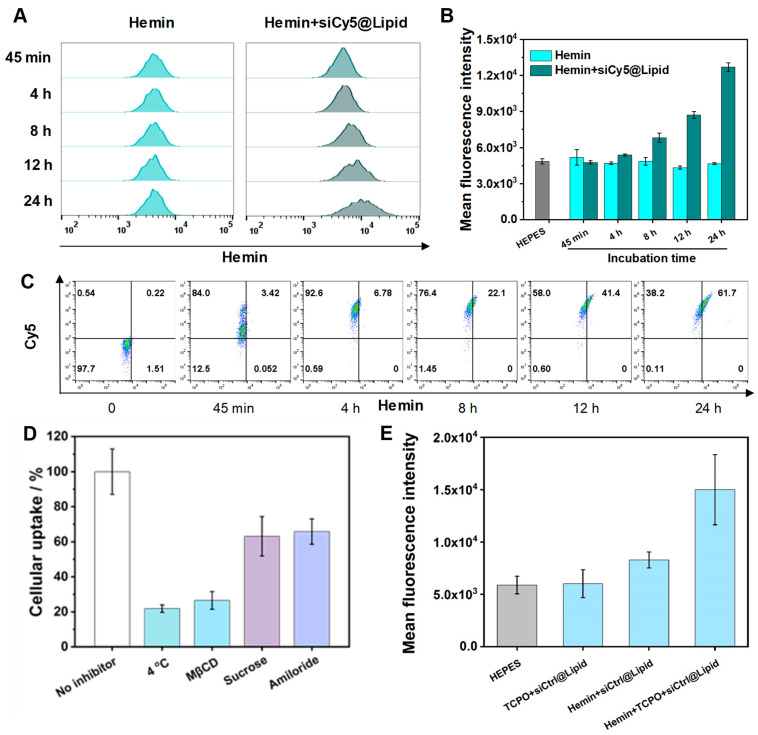
Endocytosis and cellular ROS production assays. (**A**) Investigation of hemin and Hemin+siCy5@Lipid cellular internalization after a series of incubation times. (**B**) Quantitative analysis of cellular hemin fluorescence intensity after different incubation times. (**C**) Hemin/Cy5-based cellular internalization assay in cancer cells following treatment with Hemin+siCy5@Lipid. The nanocarrier solution contained 100 ng siCy5, as well as 4 μg hemin with an N/(P+C) ratio of 9. (**D**) Endocytosis pathway study of LNP with different inhibitors. 4 °C: energy-dependent endocytosis; MβCD: lipid rafts/caveolae-mediated endocytosis; sucrose: clathrin-mediated endocytosis; amiloride: macropinocytosis. The incubation time was 4 h. Uptake was normalized to cells without an inhibitor. (**E**) Assessment of ROS production in cells exposed to various formulations. Each formulation contained siCtrl 100 ng well^−1^, TCPO 8 µg well^−1^, and hemin 130 ng well^−1^.

Excellent RNAi primarily depends on effective cellular internalization; therefore, the endocytosis of Hemin+siRNA@Lipid was explored using Cy5-labeled siRNA. The results showed that no significant signal shift was observed for naked hemin (Figure 3A). In contrast, the uptake of the Hemin+siRNA@Lipid was gradually improved after incubation for 8 h (Figure 3B). Similarly, the enhanced cytosolic delivery of siRNA through a lipid encapsulation strategy was demonstrated in Figure 3C and Figure S11 (Supporting Information). However, the hemin signal from the encapsulated group was notably lower than that of siCy5, which was attributable to the aggregation-induced quenching of photosensitizers.

In an attempt to validate the internalization pathway of the LNP, three types of inhibitors and low temperatures were adopted following literature protocols [48,49,50]. At 4 °C, energy-dependent endocytosis is affected; methyl-β-cyclodextrin (MβCD) can inhibit caveolae/lipid rafts-mediated endocytosis; sucrose is the clathrin-mediated pathway inhibitor; amiloride is utilized to interfere the Na^+^/H^+^ exchange pump in macropinocytosis. As shown in Figure 3D, low temperature and MβCD inhibited the cellular internalization of nanocarriers by 78 and 73%, respectively, demonstrating energy- and caveolae/lipid rafts-dependent endocytosis mechanisms.

Based on the good cellular uptake, we further investigated the CET-based LNP-mediated cellular ROS generation (Figure 3E). In comparison to the HEPES group, a 1.5-fold increase in the fluorescent signal was observed in the CET group. As expected, the hemin in the hydrophobic interlayer was excited by the chemical energy through the reaction of TCPO and cellular H_2_O_2_.

### 3.4. CET-Related Gene Silence Enhancement, Endosomal Escape, and Cancer Cell Killing

**Figure 4:**
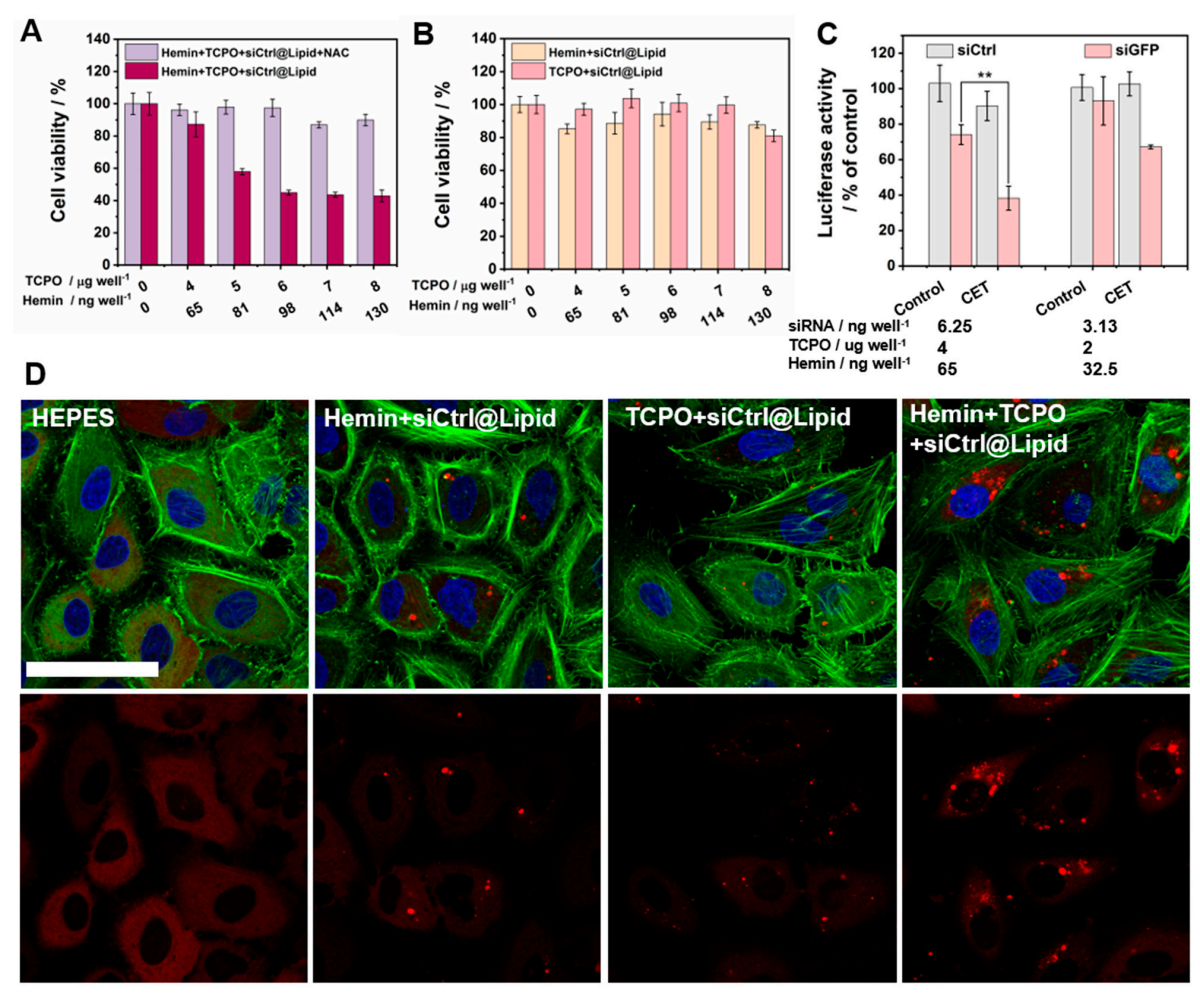
Cell viability, gene silencing, and endosomal escape assay of CET system. N/P and N/(P+C) ratios were 9. (**A**) KB cell viability after incubation with serial concentrations of Hemin+TCPO+siCtrl@Lipid with or without 3 mM NAC for 48 h. NAC was used as an antioxidant. (**B**) Cell cytotoxicity of single CET components. KB cells were incubated with LNPs for 48 h and then evaluated using an MTT assay. (**C**) Gene silence efficacy of CET-enhanced siRNA@Lipid. The CET group refers to Hemin+TCPO+siRNA@Lipid, and the control group refers to siRNA@Lipid. Luciferase activity was normalized to cells without any treatment. (**D**) CLSM of Hela mRuby3/Gal8 cells after treatment with different formulations. TCPO and hemin concentrations were the same in (**C**) on the right side; the siRNA amount was 500 ng; the incubation time was 12 h; the scale bar is 100 μm. Nuclei were stained with DAPI (blue), actin filaments were stained with rhodamine-phalloidin (green), and red fluorescence indicates Gal8-mRuby3. Statistical significances were calculated using the Student’s *t*-test. ** *p* < 0.01.

The CET-triggered ROS generation depends on the dosage and the cell type, which not only enhances siRNA delivery by destabilizing endosomal membranes but also induces cell killing. To prove the CET capability for cancer cell elimination through intracellular ROS, the cell metabolism activity was measured with or without the addition of an antioxidant (*N*-acetylcysteine, NAC). Consistent with expectations, in the absence of NAC, the cell viability exhibited a concentration-dependent reduction pattern in the presence of CET components. Notably, co-incubation with Hemin+TCPO+siRNA@Lipid for 48 h at doses of 6 µg well^−1^ TCPO and 96 ng well^−1^ hemin induced a 55% reduction in cancer cell viability. In contrast, after scavenging ^1^O_2_ with NAC, cellular metabolic activity was considerably restored, with no noticeable decrease observed (Figure 4A). Moreover, the potential cytotoxicity of individual contributors was also evaluated. Encouragingly, following a 48 h treatment of cells with Hemin+siRNA@Lipid or TCPO+siRNA@Lipid, the excellent biocompatibility of the CET fundamental elements was confirmed (Figure 4B). In addition, we demonstrated that the CET effect-mediated cancer cell killing is a time-consuming process. With a shorter incubation time, less cell lethality was detected, indicating a gradual yield of cellular ^1^O_2_ and sustained induction of cell apoptosis (Figure S12A, Supporting Information). It has been demonstrated that cancer cells manifest metabolic alterations leading to notably elevated H_2_O_2_ levels in mitochondria (50–100 μM); the overproduced H_2_O_2_ takes responsibility for CET efficacy and specificity. Human embryonic kidney 293 cells (HEK293), characterized by low H_2_O_2_ production, were employed to study the specificity of the CET system. Compared with KB cells, negligible non-selective effects were exhibited toward normal cells, even at a relatively high dose (Figure S12B, Supporting Information).

As mentioned above, the produced ROS in the CET system is beneficial for improving gene silencing via endosomal membrane lipid peroxidation. To verify this hypothesis, we measured the RNAi efficacy of LNPs with or without the CET system. In the initial step, an MTT assay was conducted for low doses of hemin and TCPO to ensure no interference with cell viability (Figure S12C, Supporting Information). Also, luciferase activity assays in KB/eGFPLuc cells showed no noticeable difference with or without the presence of the CET single factor (Figure S13A, Supporting Information). Successful improvement in gene silencing was achieved at low doses of siRNA (3.1–6.3 ng well^−1^) (Figure 4C). Subsequently, a double CET dose was introduced. As expected, high RNAi efficacy was attained at a low siRNA dose of 3.1 ng, which was explainable by the ^1^O_2_ assistance in endosomal membrane disruption (Figure S13B, Supporting Information).

To further confirm this conclusion, an endosomal escape reporter cell line, HeLa cells expressing Gal8-mRuby3, was employed to assess the endosomal rupture capability of the CET system (Figure 4D). In this model, a cytosolic Gal8-mRuby3 fusion protein would be recruited to disrupted endosomal membranes, forming visible bright fluorescent spots [44,45]. In this model, the molecular targets of Gal8 are glycans containing galactose, found exclusively on the inner membrane of endosomes. Following a 12 h treatment, formulations combining hemin and TCPO were found to prompt endosomolytic events compared with their individual counterparts, showing to be nearly threefold (*p* value of 0.03) and 10-fold (*p* value of 0.02) higher than the TCPO and hemin group, respectively (Figure S14A, Supporting Information). Meanwhile, no intracellular punctate spot was detected in the HEPES group. The endosomal escape event started at approximately 4 h of incubation and extended for over 48 h (Figure S14B,C, Supporting Information). Altogether, consistent with the conclusion of Figure 2E, Figure S8, and Figure 3E, the designed CET system could induce effective double bond oxidation in phospholipid through lipid peroxidation, providing an opportunity for nucleic acid to escape from LNP and endosome.

### 3.5. CET-Enhanced Cancer Cell Killing

**Figure 5:**
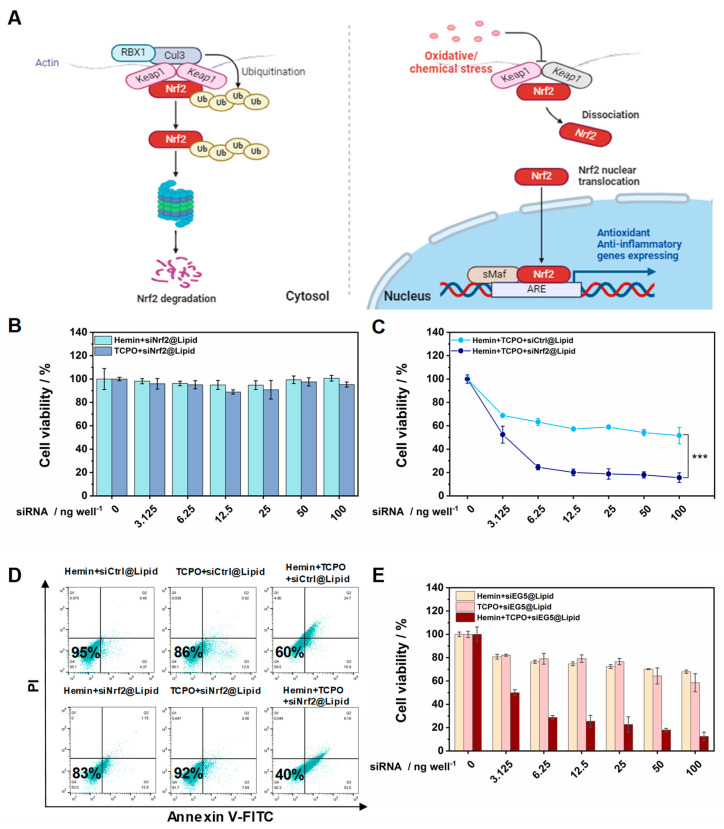
CET-enhanced cancer cell-killing effect. (**A**) Schematic illustration of transcription factor Nrf2–Keap 1 system-associated detoxification. (**B**) Assessment of KB cell viability following exposure to various concentrations of Hemin+siNrf2@Lipid and TCPO+siNrf2@Lipid. Incubation time was 48 h. (**C**) siNrf2-based LNPs for killing KB cells. The TCPO amount was 8 µg well^−1^, and the hemin amount was 130 ng well^−1^. (**D**) Annexin V-FITC/PI cell apoptosis analysis of KB cells after different treatments. The siRNA amount was 1.88 µg well^−1^; the TCPO amount was 60 µg well^−1^; the hemin amount was 975 ng well^−1^; the incubation time was 48 h. (**E**) Dose titration of CET system-assistant siEG5-based LNPs. The TCPO amount was 8 µg well^−1^, and the hemin amount was 130 ng well^−1^. Incubation time was 48 h. Statistical significance was calculated using the Student’s *t*-test. *** *p* < 0.001.

As demonstrated in Figure 4, the CET system at a standard dose (4 µg TCPO and 65 ng hemin) was capable of delivering siRNA and holding good biocompatibility; at a high dose (8 µg TCPO and 130 ng hemin), more ROS can be produced for directly killing cancer cells. To enhance the anticancer capability of CET toward cancer cells, gene silencing of the Nrf2 (also known as Nfe2l2)–Keap1 system was introduced; it is considered a master transcriptional regulator of endogenous oxidative stress [51,52,53,54,55,56]. For ROS-mediated treatments, Nrf2 plays a crucial role in helping cancer cells evade oxidative damage. Under oxidative stress, Nrf2 dissociates from Keap1, relocates to the nucleus, and takes shape into a heterodimer with its partner (Figure 5A, right). The resulting complex binds to antioxidant-responsive element sequences, activating downstream genes and antioxidants. Oppositely, under healthy conditions, Nrf2 mRNA is constitutively expressed and bound to its inhibitor, Keap1, which facilitates the binding of the Cullin-3 (Cul3)/RING box protein1 (RBX1) E3 ubiquitin ligase complex, consequently causing the proteasomal degradation of Nrf2 (Figure 5A, left). By adopting the Nrf2-Keap1 pathway as the target, we used Nrf2 siRNA (siNrf2) to synergize with the ROS oxidative effect, and the generated ROS enhanced the siRNA endosomal escape simultaneously. After incubating cancer cells with Hemin+siNrf2@Lipid or TCPO+siNrf2@Lipid for 48 h, the excellent biocompatibility of the prepared siNrf2-synergized CET system was validated (Figure 5B). Opposite to the control groups, after integrating hemin and TCPO, more than 80% of cells were dead at 8 µg well^−1^ TCPO, 130 ng well^−1^ hemin, and 12.5 ng siRNA doses, and a 39% reduction was demonstrated compared with Hemin+TCPO+siCtrl@Lipid (Figure 5C). Consistent with the conclusion in Figure S12B, after replacing siCtrl with siNrf2, the CET system maintained good biosafety to normal cells (Figure S15, Supporting Information). Next, with the help of Annexin V-FITC and PI, the apoptosis rate of KB cells incubated with various formulations was determined via flow cytometry (Figure 5D). The apoptosis level of cells incubated with the CET group in the presence of siNrf2 was around 60%, significantly surpassing that of the CET group alone (36%). Further evolution of Nrf2 gene silencing at the mRNA level was conducted through RT-qPCR assay. As shown in Figure S16A, treatments with all LNPs including siNrf2 induced the significant knockdown of Nrf2 mRNA (46% by Hemin+siNrf2@Lipid, 51% by TCPO+siNrf2@Lipid, and 87% by Hemin+TCPO+siNrf2@Lipid); conversely, siCtrl groups maintained expression levels comparable to those of the HEPES (20 mM, pH 7.4) group. Moreover, the Nrf2 mRNA expression level of the CET group was much lower than that of the corresponding control groups. These results proved that the CET involvement results in improved gene silencing efficacy at the mRNA expression level.

In addition, we underscored the CET-enhanced RNAi using an alternative type of siRNA. Eglin 5 (EG5/KSP), a motor protein that provides a crucial role in cellular mitosis, can be silenced to impede the organization of the mitotic spindle apparatus, leading to cell-cycle arrest, therefore initiating programmed cellular apoptosis in tumor cells [39,57]. As shown in Figure 5E, the survival rate of cells incubated with siEG5 was obviously decreased compared with Hemin+siEG5@Lipid and TCPO+siEG5@Lipid. Notably, the highest cell-killing efficacy, around 87%, was achieved at a dose of 100 ng siEG5, 8 µg TCPO, and 130 ng hemin. The apoptosis assay also proved superior CET-improved siEG5 cell killing, with a total apoptotic cell percentage of 68%, which is much higher than those for a single treatment (Figure S17, Supporting Information). The improvement of gene silencing using CET was also demonstrated at the mRNA level (Figure S16B, Supporting Information). The results demonstrated that the incorporation of CET into the LNP amplified the effectiveness of therapeutic gene silencing.

## 4. Conclusions

Inspired by the photochemical internalization technology for enhanced cytosolic nucleic acid delivery [30,31,32,33,34,35,36,37], we first designed LNPs containing siRNA and CET components (Hemin+TCPO+siRNA@Lipid) for enhanced chemical internalization excited by H_2_O_2_ within the tumor environment. Co-encapsulating the hydrophobic CET donor TCPO and acceptor hemin facilitated their energy transfer, subsequently exciting O_2_ to produce ^1^O_2_. Our investigation aimed to answer the following questions: (i) Can CET-triggered ROS generation enhance the endosomal escape of siRNA@Lipid? (ii) Can the ROS also directly damage cancer cells? (iii) Can combined silencing of tumor-protective Nrf2 enhance ROS-induced cancer cell killing? All the three questions were answered. The CET process boosted endosomal disruption as measured by a Gal8 sensor. Three distinct types of siRNA, siGFP, siEG5, and siNrf2, were employed to verify CET-assisted gene silencing. A relatively high RNAi efficacy was attained at a low siGFP at around 3–6 ng per well. For siEG5, the induction of the apoptosis of cancer cells was demonstrated, achieving cell-killing efficacy of approximately 75% at 12.5 ng. Moreover, a 24% improvement in apoptosis level was observed through the regulation of endogenous oxidative stress by siNrf2. Our research underscores the great promise of CET-enhanced LNPs for advancing antitumoral nucleic acid therapeutics.

## Data Availability

The data supporting the findings of this study are accessible within the article and its Supporting Information files.

## Supplementary Materials

The following supporting information can be downloaded at https://www.mdpi.com/article/10.3390/pharmaceutics16060779/s1, Figure S1: Chemical structure and MALDI-MS spectrum of the ionizable lipopeptide tetra-oleoyl-tri-lysino-succinoyl tetraethylene pentamine; Figure S2: Illustration of the generation of CET-enhanced LNPs via rapid mixing; Figure S3: Illustration depicting the chemical reaction of TCPO and H_2_O_2_ and the excitation of hemin from the ground singlet state to the excited singlet state through energy transfer with 1,2-dioxetanedione; Figure S4: Degradation spectra of DPA with different conditions; Figure S5: Particle diameter and ROS generation properties of LNPs with different TCPO/hemin ratios; Figure S6: UV–vis light absorption spectra of different formulations; Figure S7: Loading determination of hemin and TCPO; Figure S8: PDI of siRNA@Lipid and Hemin+TCPO+siRNA@Lipid with or without the addition of H_2_O_2_; Figure S9: CET-enhanced LNPs stability against varying concentrations of NaCl and heparin; Figure S10: Gene silencing efficiency in DU145/eGFPLuc and N2a/eGFPLuc cells treated with LNPs at siRNA amounts; Figure S11: Endocytosis of LNPs; Figure S12: Cell viability of KB cells incubated with LNPs; Figure S13: Gene silencing efficiency of KB/eGFPLuc treated with various LNPs; Figure S14: Endosomal escape assay; Figure S15: Cytocompatibility of Hemin+TCPO+siNrf2@Lipid in H_2_O_2_ low producing cells; Figure S16: Analysis of gene silencing at mRNA expression level by RT-q PCR; Figure S17: Cell apoptosis assay; Table S1: Molar ratio of each compound for LNPs with different N/P ratios.
