# Supplementary material for: Dual Effect by Chemical Electron Transfer Enhanced siRNA Lipid Nanoparticles: Reactive Oxygen Species-Triggered Tumor Cell Killing Aggravated by Nrf2 Gene Silencing
Source: Pharmaceutics. 2024 Jun 7;16(6):779. doi: 10.3390/pharmaceutics16060779 (PMC11207527; doi:10.3390/pharmaceutics16060779)
Supplement: Supplementary file 1 [file pharmaceutics-16-00779-s001.zip › pharmaceutics-3013027-supplementary.pdf]

Supporting Information

Dual Effect by Chemical Electron Transfer Enhanced siRNA Lipid Nanoparticles: Reactive Oxygen Species-Triggered Tumor Cell Killing Aggravated by Nrf2 Gene Silencing

Fengrong Zhang[1]*, Tobias Burghardt[1], Miriam Höhn[1], and Ernst Wagner[1,2,3]*

[1] Pharmaceutical Biotechnology, Center for Nanoscience, Ludwig-Maximilians-Universität (LMU) Munich, 81377 Munich, Germany; tobias.burghardt@cup.uni-muenchen.de (T.B.); miriam.hoehn@cup.uni-muenchen.de (M.H.)

[2] CNATM-Cluster for Nucleic Acid Therapeutics Munich, 81377 Munich, Germany

[3] Center for Nanoscience (CeNS), LMU Munich, 81377 Munich, Germany

*Correspondence: fengrong.zhang@cup.uni-muenchen.de (F.Z.); ernst.wagner@cup.uni-muenchen.de (E.W.)

Experimental Section

Physicochemical Characterization. The hydrodynamic size, polydispersity index (PDI), and zeta potential of lipid nanoparticles (LNPs) were tested with a Zetasizer Nano ZS (Malvern Instruments, UK). LNPs containing 1 μg of siRNA in a total volume of 200 μL were diluted 1:5 with 20 mM HEPES buffer (pH 7.4) before measurement using a folded capillary cell (DTS1070). For size measurements, each sample underwent three times with 10 subruns. Zeta potentials were determined by the Smoluchowski equation, with three measurements (15 subruns), and data were analyzed using Zetasizer software (version 7.13). The ultraviolet-visible (UV-vis) light absorption spectra of hemin and bis(2,4,6-trichlorophenyl) oxalate (TCPO) in different concentrations were measured using a Cary 3500 UV–Vis spectrophotometer (Agilent Technologies, Palo Alto, CA, USA). A matrix-assisted laser desorption/ionization mass spectrometer (MALDI-MS, Bruker Daltonics, Bremen, Germany) was used to characterize the ionizable lipopeptide. A JEM 1011 transmission electron microscope (TEM, JEOL, Tokyo, Japan) at 80 kV acceleration voltage was used to analyze the morphology of LNPs. First, carbon-coated TEM copper grids (300 mesh, 3.0 mm OD; Ted Pella, Redding, CA, USA) underwent hydrophilization using mild argon plasma (420 V, 1 min). After that, 10 μl sample droplets were dropped on the grids and incubated for 3 min. Following incubation, the solution was removed, and the grid was washed once before being stained with 10 μl of 1% uranyl acetate solution for 5 seconds. Finally, the excess staining solution was removed, and the grid was allowed to air dry for 20 min before being measured by TEM.

Gel Shift Assay. A 2.5% (w/w) agarose gel solution containing GelRed was prepared for the siRNA binding assay. Agarose was dissolved in TBE buffer by boiling and cooling to approximately 50 °C. GelRed (1/1000, v/v) was added to the solution. After adding 4 μL loading buffer, LNPs (containing 100 ng siRNA in 20 μl HEPES buffer) with different N/P ratios were loaded into the preprepared wells. Electrophoresis was conducted at 120 V

for 40 min, and subsequently, the gel was photographed using a Dark Hood DH-40 UV transilluminator from Bio-step (Burkhardtsdorf, Germany).

Ribogreen Assay. siRNA loading efficacy was determined by a Quant-iTTM RiboGreen® RNA assay kit. 50 μL LNPs were mixed with 50 μL of TE buffer or TE buffer containing 2% Triton X-100, and added to a 96-well plate. Next, 100 μL of Ribogreen solution was added to each well and incubated at 37 °C for 15 min. The fluorescence intensity was detected using a microplate reader (Tecan, Männedorf, Switzerland). The fluorescence intensity from TE buffer treated LNPs (F_{TE}) represents unencapsulated siRNA, and fluorescence intensity from 2% Triton X-100 treated LNPs (F_{TX}) represents total siRNA. The siRNA encapsulation efficiency can be determined as

$$Encapsulation\,\% = \frac{F_{TX}-F_{TE}}{F_{TX}} \times 100\%$$

LNPs Stability Assay Under Different Ionic Strength Conditions. 50 μL of TE buffer containing varying concentrations of Heparin and NaCl were added to 96-well plates, respectively. Subsequently, 50 μL of LNPs with an N/(P+C) of 9, containing 65 ng hemin, 4 μg TCPO, and 50 ng siRNA were added to each well. Final NaCl concentrations ranged from 0 to 5 M, and the final amounts of heparin ranged from 0 to 5 IU per μg of siRNA. After incubating for 30 min at 37 °C, 100 μL of Ribogreen solution was added to each well and incubated at 37 °C for 15 min. The fluorescence intensity was measured using a microplate reader. Additionally, the fluorescence intensity of LNPs mixed with 2% Triton X-100 and TE buffer-treated LNPs was also tested. The fluorescence intensity of LNP samples (F_S) treated with different ionic strengths represents leaked siRNA. The fraction of dye exclusion can be calculated as

$$Fraction\ of\ dye\ exclusion = 1 - \frac{\mathrm{F}_{S}-\mathrm{F}_{TE}}{\mathrm{F}_{TX}-\mathrm{F}_{TE}}$$

Cell Culture. Human cervix carcinoma cells KB, Hela, human embryonic kidney cells HEK293, human prostate cancer cells DU145, and murine neuroblastoma cells N2a were obtained from DSMZ (German Collection of Microorganisms and Cell Cultures, Braunschweig, Germany). KB cells, DU145 cells, and N2a cells stably transfected with the eGFP-luciferase gene, KB/eGFPLuc, DU145/eGFPLuc, N2a/eGFPLuc, were used in gene silencing efficiency assay [40,43]. HeLa cells stably expressing galectin 8 (Gal8)-mRuby3 fusion protein were chosen to demonstrate endosomal escape [44,45]. The PB-CAG-mRuby3-Gal8-P2A-Zeo plasmid was a gift from Jordan Green's lab (Addgene plasmid no. 150815; http://n2t.net/addgene:150815; RRID: Addgene_150815). KB, KB/eGFPLuc, HeLa-Gal8-mRuby3, N2a, and N2a/eGFPLuc were cultured in Dulbecco's modified Eagle's medium (DMEM) at 37 ^{o}C with 5% CO_2. HEK293 cells were maintained in low-glucose DMEM. DU145 and DU145/eGFPLuc were cultured in Roswell Park Memorial Institute (RPMI)-1640 medium. The Dulbecco's modified Eagle's medium (DMEM), low-glucose DMEM, and RPMI-1640 medium were supplemented with 10 % fetal bovine serum, 100 U mL^{-1} penicillin, and 100 μg mL^{-1} streptomycin.

Reactive Oxygen Species (ROS) Generation Study. For the analysis of ROS generation, three different formulations were prepared (TCPO+siRNA@Lipid, Hemin+siRNA@Lipid, and Hemin+TCPO+siRNA@Lipid). Each formulation contained siCtrl 100 ng $well^{-1}$, TCPO 8 μg $well^{-1}$, and hemin 130 ng $well^{-1}$. First, KB cells were seeded in 96-well plates at a density of 10000 cells/well one day before the treatments. Thereafter, cells were incubated with the LNPs for 48 h. Then, cells were washed with PBS, and a medium containing 750 nM CellROX Green reagent was added. After incubation for 45 min at 37 ^{o}C, cells were collected and measured by the flow cytometer (Beckman Coulter, Fullerton, CA, USA). The data were analyzed using FlowJo 7.6.5.

Endocytosis Pathway Assay. One day before the treatments, KB cells were plated in 96-well plates at a density of 10000 cells/well. Subsequently, the medium was replaced with 100

μL of serum-free medium containing various inhibitors (methyl-β-cyclodextrin 5 mM, sucrose 450 mM, amiloride 100 μM) and incubated for 1 h at 37 °C. Afterwards, the medium was replaced with 80 μL of fresh medium (containing 10 % fetal bovine serum, 100 U mL^{-1} penicillin, and 100 μg mL^{-1} streptomycin) and 20 μL of buffer containing 100 ng siCy5 and 4 μg hemin. For the 4 °C treatment, cells were placed at 4 °C cooling room for 1 h before adding siCy5-containing LNP in the ice-cold medium. Finally, the cells were collected and measured by a flow cytometer.

Cell Viability Assay. One day before the treatments, KB cells were seeded in 96-well plates at a density of 5000 cells/well. Subsequently, cells were incubated with a serial concentration of LNPs for 24 h or 48 h. Then standard MTT assay was carried out following the protocol. In the case of the H_2O_2 low-producing cell assay, HEK293 cells were used, with an incubation time of 48 h, and the LNP was Hemin+TCPO+siRNA@Lipid. For the anti-oxidation experiment, KB cells were co-incubated with Hemin+TCPO+siRNA@Lipid and 3 mM *N*-acetylcysteine for 48 h. Finally, cell viability was measured as described above.

siEG5 Combined CET System for Cancer Cell Killing. To measure the enhanced gene silencing efficacy, siEG5 was incorporated into the CET system. KB cells were seeded in 96 plates at a density of 5000 cells/well 24 h prior to the treatments. Then, the medium was replaced with 80 μL of fresh medium (containing 10 % fetal bovine serum, 100 U mL^{-1} penicillin, and 100 μg mL^{-1} streptomycin) and 20 μL of LNPs in HEPES buffer at various concentrations was added to each well. The LNPs included Hemin+siEG5@Lipid, TCPO+siEG5@Lipid, and Hemin+TCPO+siEG5@Lipid. After incubation for 48 h, a MTT assay was performed to analyze the cell viability.

siNrf2 Combined CET System for Cancer Cell Killing. KB cells (5000 per well) were seeded in 96-well plates in 100 μL of medium 24 h prior to the treatment. Then, the medium was replaced with 80 μL of fresh medium (containing 10 % fetal bovine serum, 100 U mL^{-1}

penicillin, and 100 μg mL^{-1} streptomycin), and 20 μL of HEPES buffer containing LNPs. The TCPO amount was 8 μg $well^{-1}$ and the hemin amount was 130 ng $well^{-1}$. After incubation for 48 h, a MTT assay was performed.

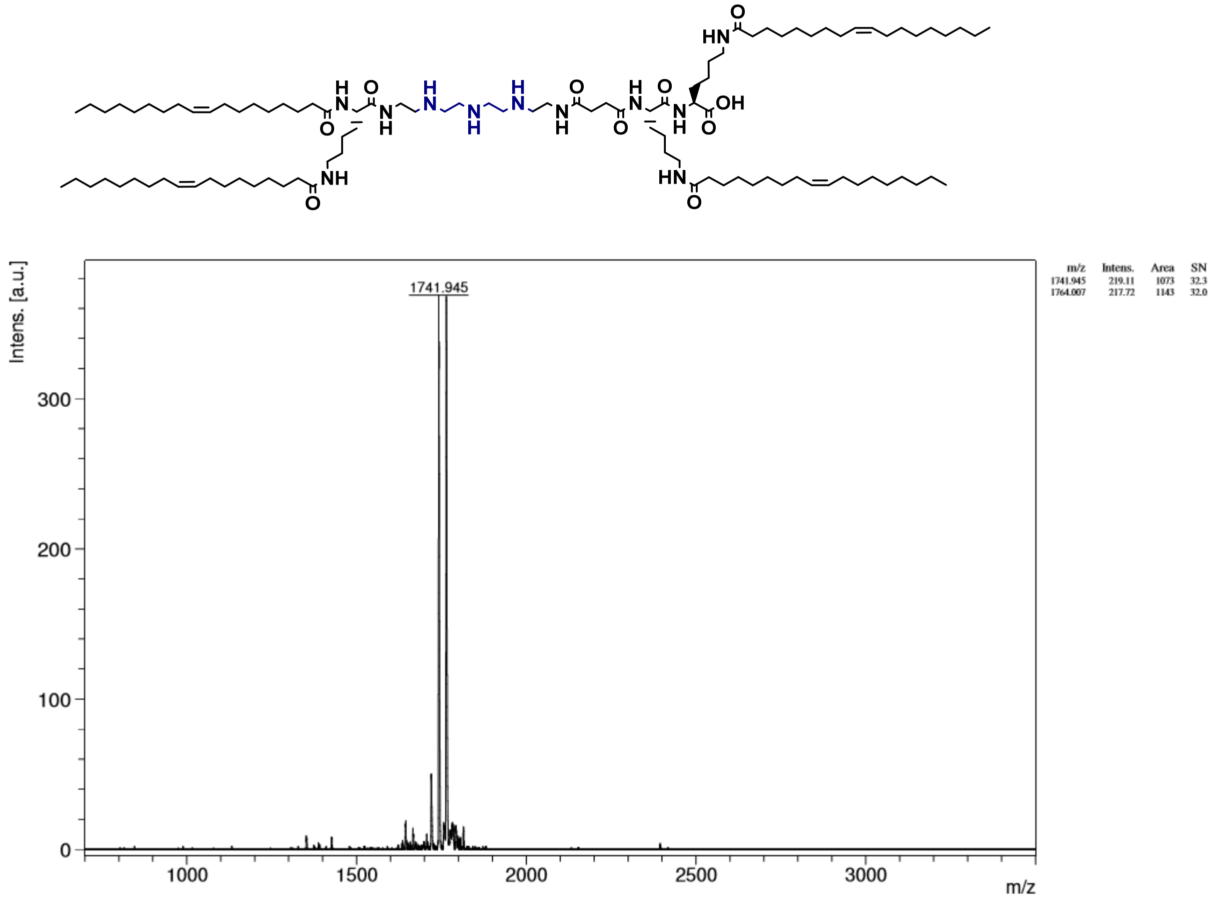

Figure S1. Chemical structure and MALDI-MS spectrum of the ionizable lipopeptide tetra-oleoyl-tri-lysino-succinoyl tetraethylene pentamine. Molecular weight:1730.48. $[M+NH_4]^+$ found 1741.95 and $[M+K]^+$ found 1764.00.

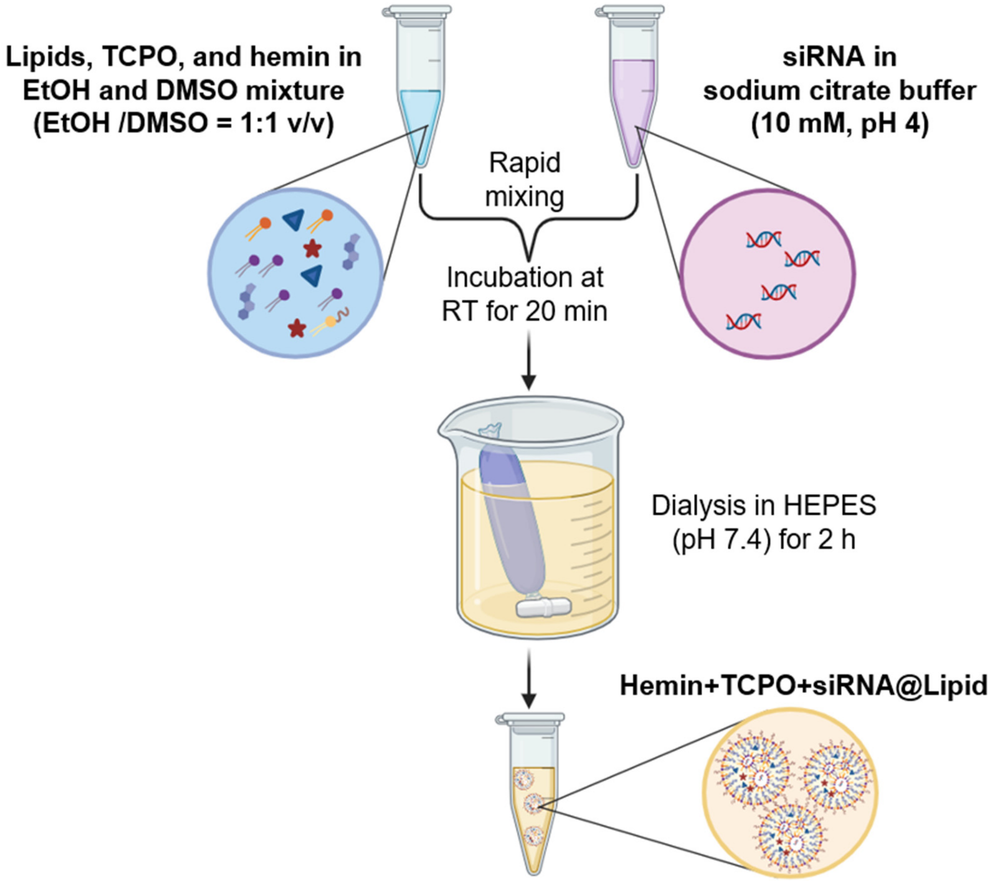

Figure S2. Illustration of the generation of chemical electron transfer (CET)-based LNPs via rapid mixing. Hemin and TCPO for the CET effect were dissolved in DMSO, and lipids were dissolved in ethanol. The ionizable lipopeptide enables siRNA encapsulation through its positive charge at the low pH of the sodium citrate buffer (pH 4).

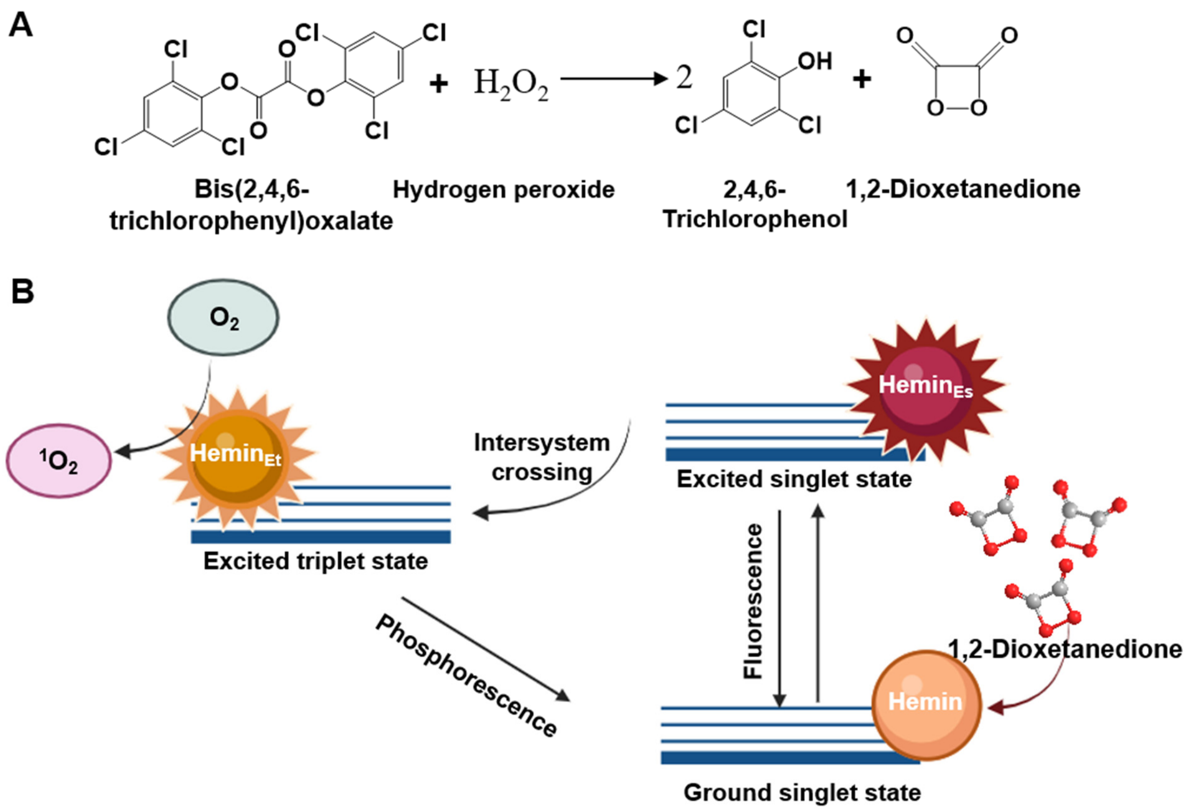

Figure S3. Illustration depicting (A) the chemical reaction of TCPO and H_2O_2 and (B) the excitation of hemin from the ground singlet state to the excited singlet state through energy transfer with 1,2-dioxetanedione. Subsequently, through intersystem crossing, it transitions to an excited triplet state which is capable of reacting with oxygen to generate $^1O_2$.

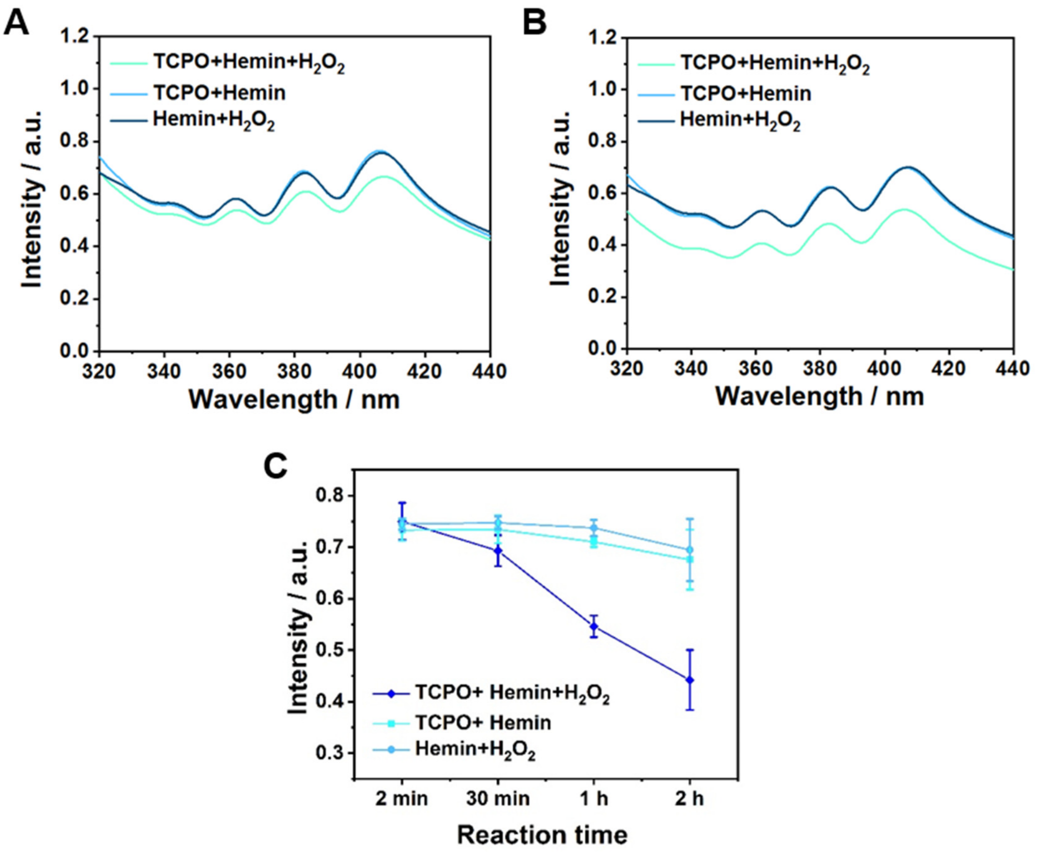

Figure S4. Degradation spectra of 9,10-diphenanthraquinone (DPA) with different conditions. (A) T = 30 min and (B) T = 1 h. (C) UV–vis absorption intensity of DPA at 383 nm over reaction time. The concentrations of DPA, TCPO, and hemin used in DPA degradation assay were 15 μg mL^{-1}, 0.17 mg mL^{-1}, and 0.78 μg mL^{-1}, respectively.

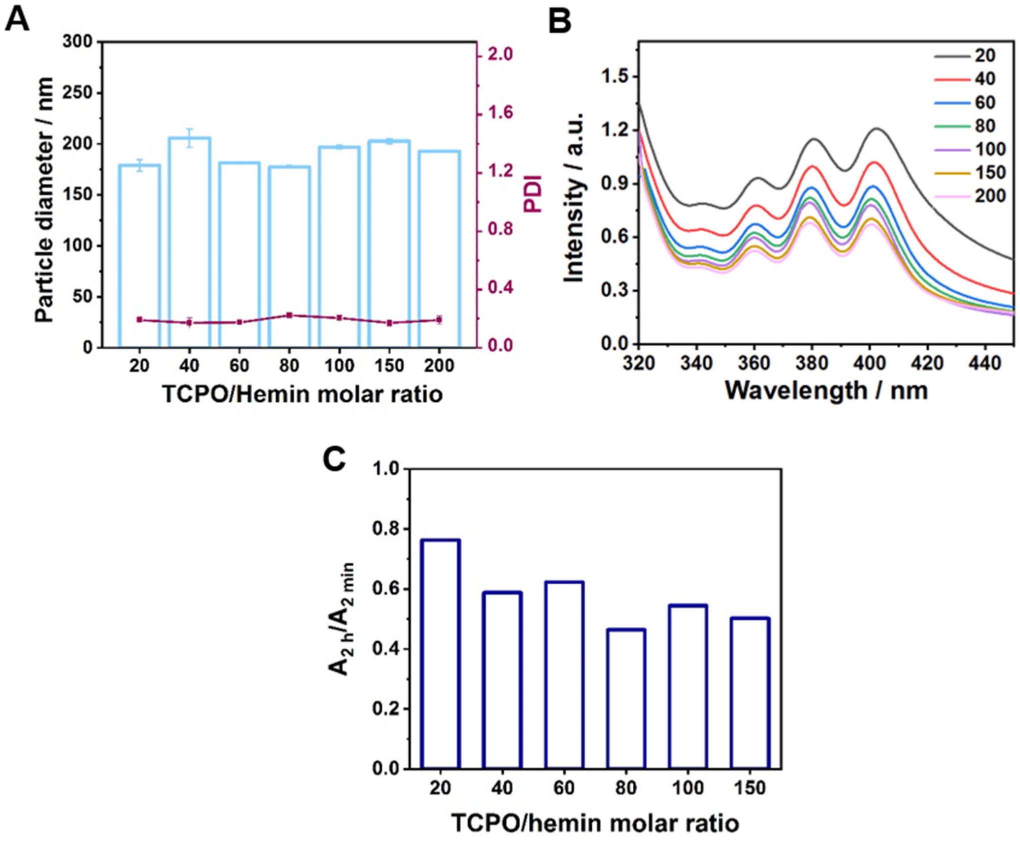

Figure S5. Particle diameter and ROS generation properties of LNPs with different TCPO/hemin ratios. (A) Size distributions and PDI of Hemin+TCPO+siRNA@Lipid. The N/(P+C) ratio was 9, the siRNA amount was 1 μg, the hemin amount was 1.3 μg, and the TCPO amount was 80 μg. (B) Degradation spectra of DPA after adding different LNPs with different TCPO/hemin ratios. The concentration of H_2O_2 adopted was 5 mM. T = 2 min. (C) Absorption intensity ratio of DPA at 383 nm between reaction times of 2 h and 2 min for different LNPs. The N/(P+C) ratio was 3, the siRNA amount was 1 μg, and the TCPO amount was 80 μg. The molar ratio of different lipids was 50/38.5/10/1.5 (ionizable lipopeptide/cholesterol/phospholipid/DMG-PEG).

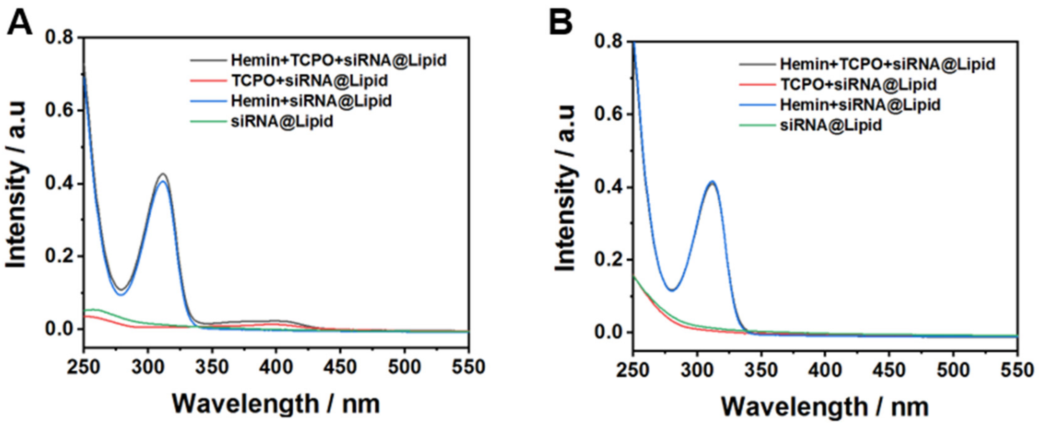

Figure S6. UV–vis light absorption spectra of different formulations. Hemin+TCPO+siRNA@Lipid, TCPO+siRNA@Lipid, Hemin+siRNA@Lipid, and siRNA@Lipid (A) before and (B) after adding H_2O_2.

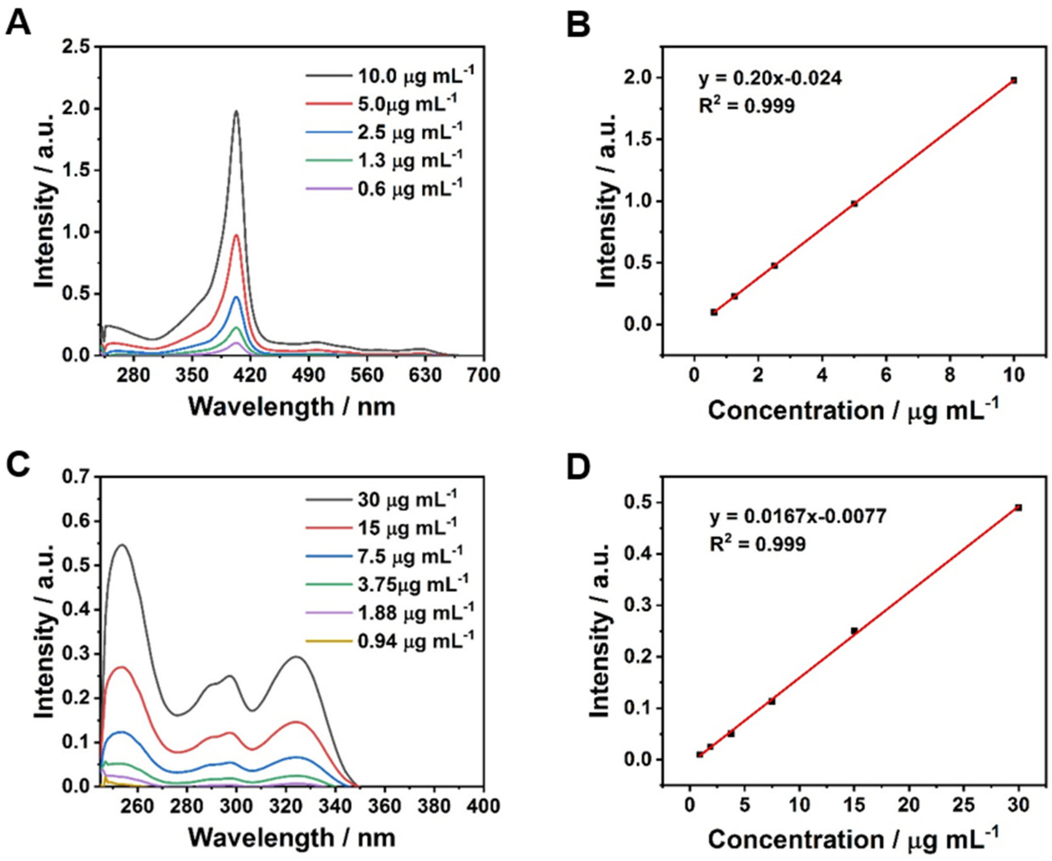

E

N/(P+C)	Hemin loading efficiency	TCPO loading efficiency
**3**	58.2%	14.2%
**4.5**	53.1%	12.1%
**9**	62.8%	24.2%
**18**	57.6%	23.0%

Figure S7. Loading determination of hemin and TCPO. UV–vis light absorption spectra of (A) hemin and (C) TCPO with different concentrations. Curve fitting for (B) hemin and (D) TCPO absorption. (E) Loading efficiencies of hemin and TCPO in optimized CET-enhanced LNPs with N/(P+C) ratios of 3.0, 4.5, 9, and 18.

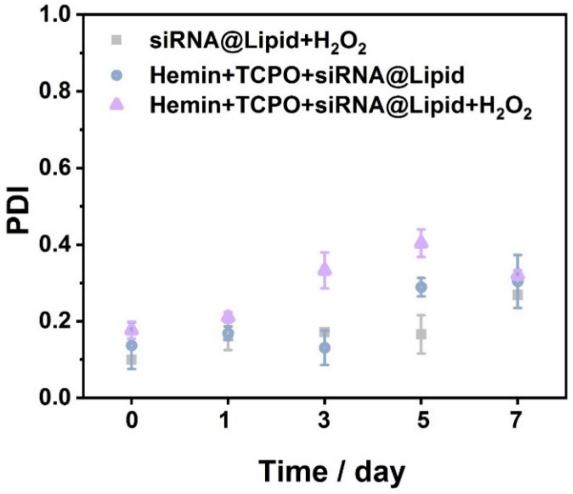

Figure S8. PDI of siRNA@Lipid and Hemin+TCPO+siRNA@Lipid with or without the addition of H_2O_2. The siRNA amount was 1 μg, the hemin amount was 1.3 μg, and the TCPO amount was 80 μg. LNPs were incubated in PBS (pH 7.4) at 37 ^{o}C for 1 week.

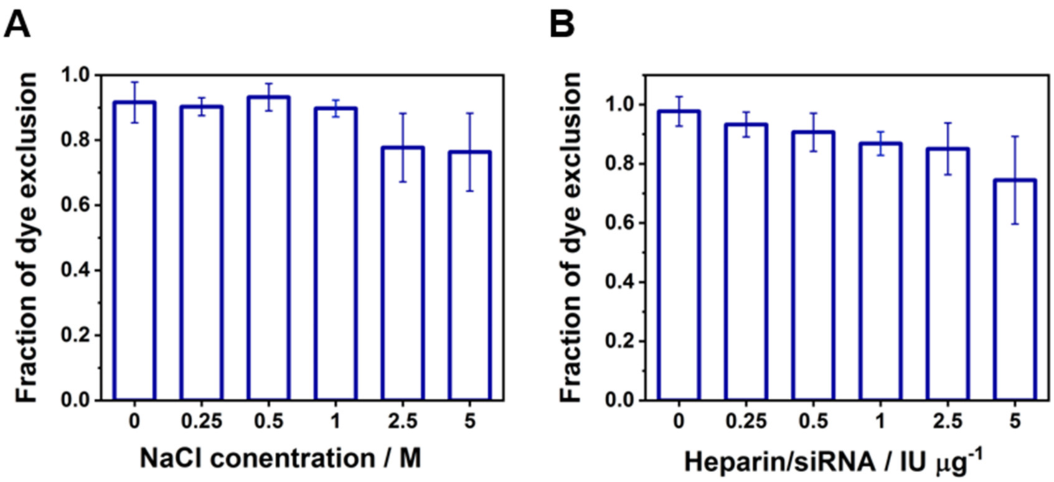

Figure S9. CET-enhanced LNPs stability against varying concentrations of (A) NaCl and (B) heparin. Ribogreen was utilized to detect free siRNA. The formulations contained siCtrl 1 μg, TCPO 80 μg, and hemin 1.3 μg.

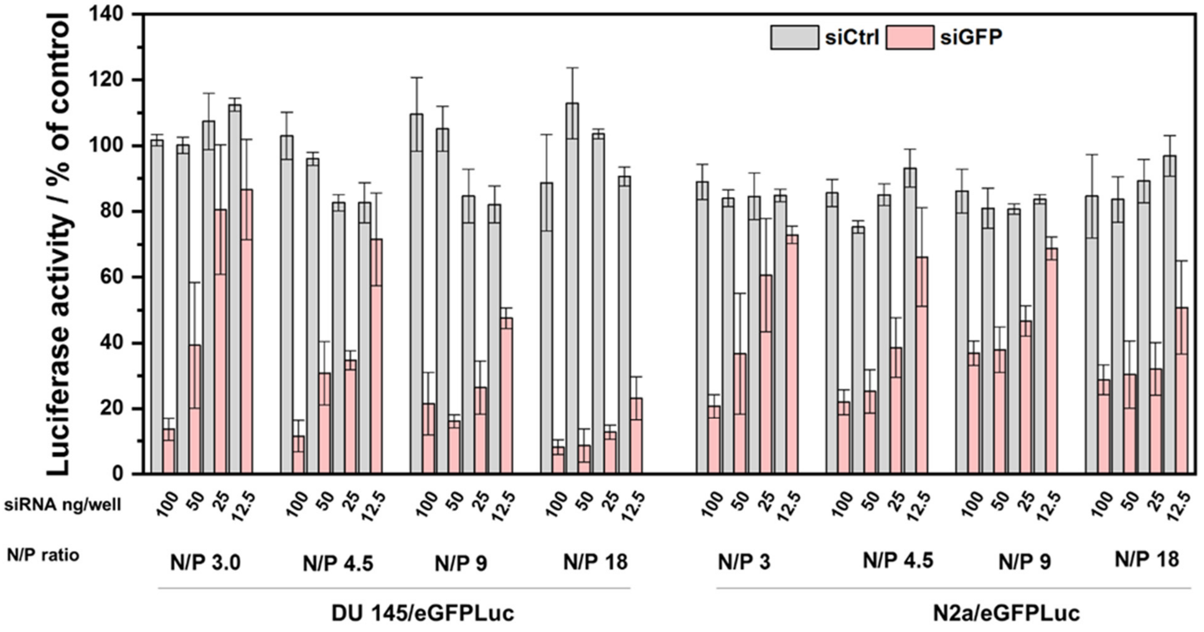

Figure S10. Gene silencing efficiency in DU145/eGFPLuc and N2a/eGFPLuc cells treated with LNPs at siRNA amounts of 12.5, 25, 50, and 100 ng for 48 h. Luciferase activity was normalized to cells without any treatment.

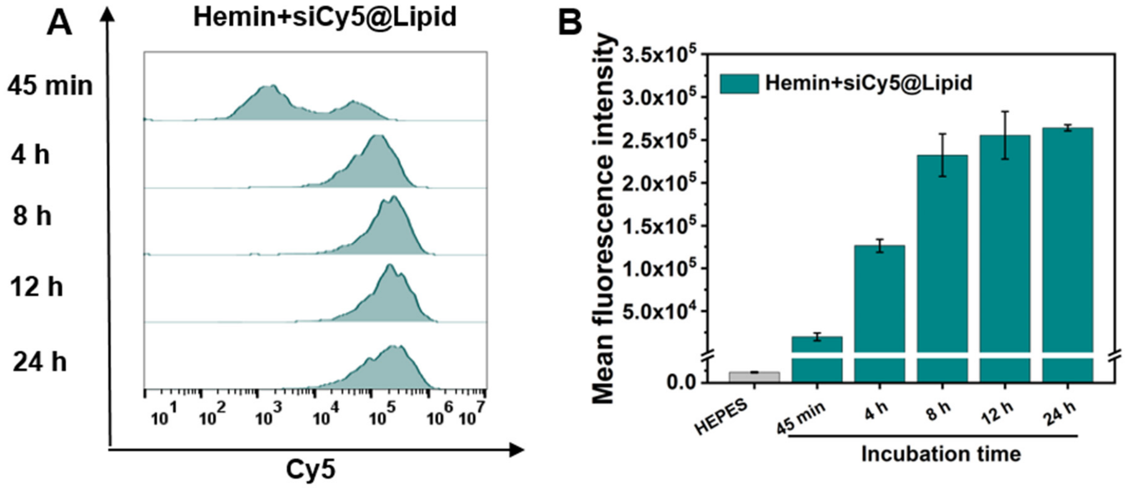

Figure S11. Endocytosis of LNPs. (A) Cellular uptake study of Hemin+siCy5@Lipid after 45 min, 4 h, 8 h, 12 h, and 24 h of incubation. The 20% siRNA was labelled with Cy5. (B) Quantitative data of cellular Cy5 fluorescence intensity after varying incubation time. The nanocarrier solution contained 100 ng siCy5 as well as 4 μg hemin with an N/(P+C) ratio of 9.

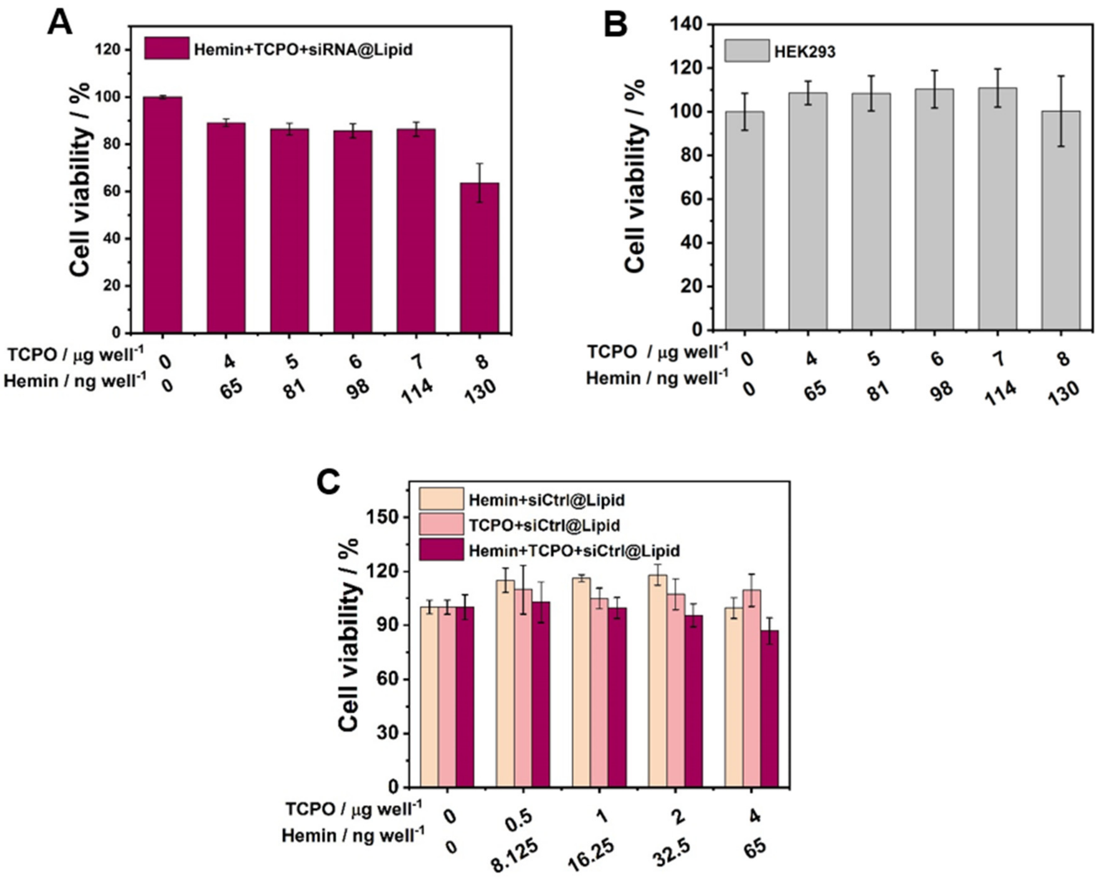

Figure S12. Cell viability of KB cells incubated with LNPs. N/P and N/(P+C) ratios were 9. (A) Cells were treated with serial concentrations of Hemin+TCPO+siRNA@Lipid for 24 h. (B) Cytocompatibility of Hemin+TCPO+siCtrl@Lipid in H_2O_2 low producing cells (HEK293). Incubation time was 48 h. (C) Cell viability of KB cells incubated with serial concentrations of Hemin+siRNA@Lipid, TCPO+siRNA@Lipid, and Hemin+TCPO+siRNA@Lipid. Incubation time was 48 h.

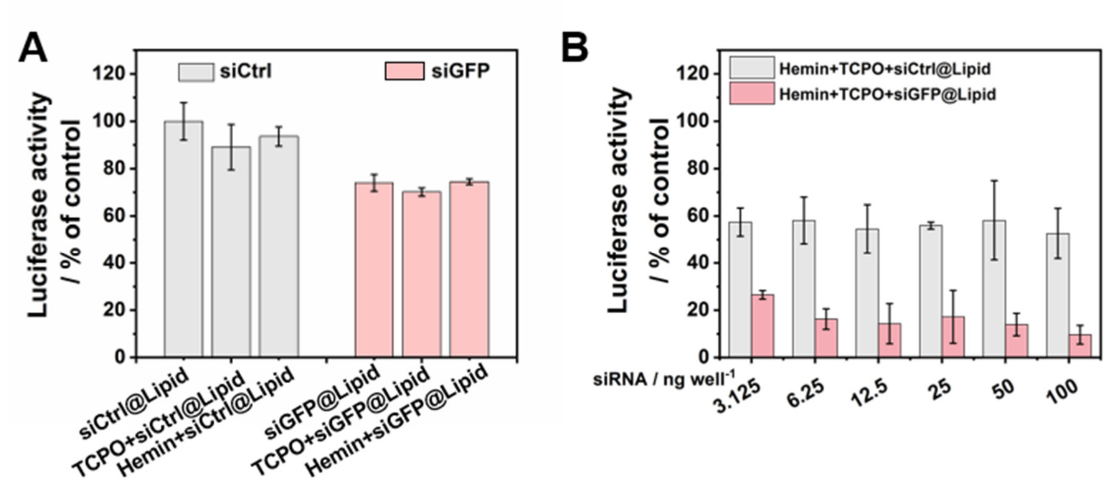

Figure S13. Gene silencing efficiency of KB/eGFPLuc treated with various LNPs. (A) Cells were treated with control LNPs for 48 h. The siRNA amount was 6.25 ng well^{-1}; the TCPO amount was 4 μg well^{-1}; the hemin amount was 65 ng well^{-1}. (B) Gene silencing efficiency of siRNA@Lipid enhanced with a higher CET dose (8 μg TCPO well^{-1}; 130 ng hemin well^{-1}).

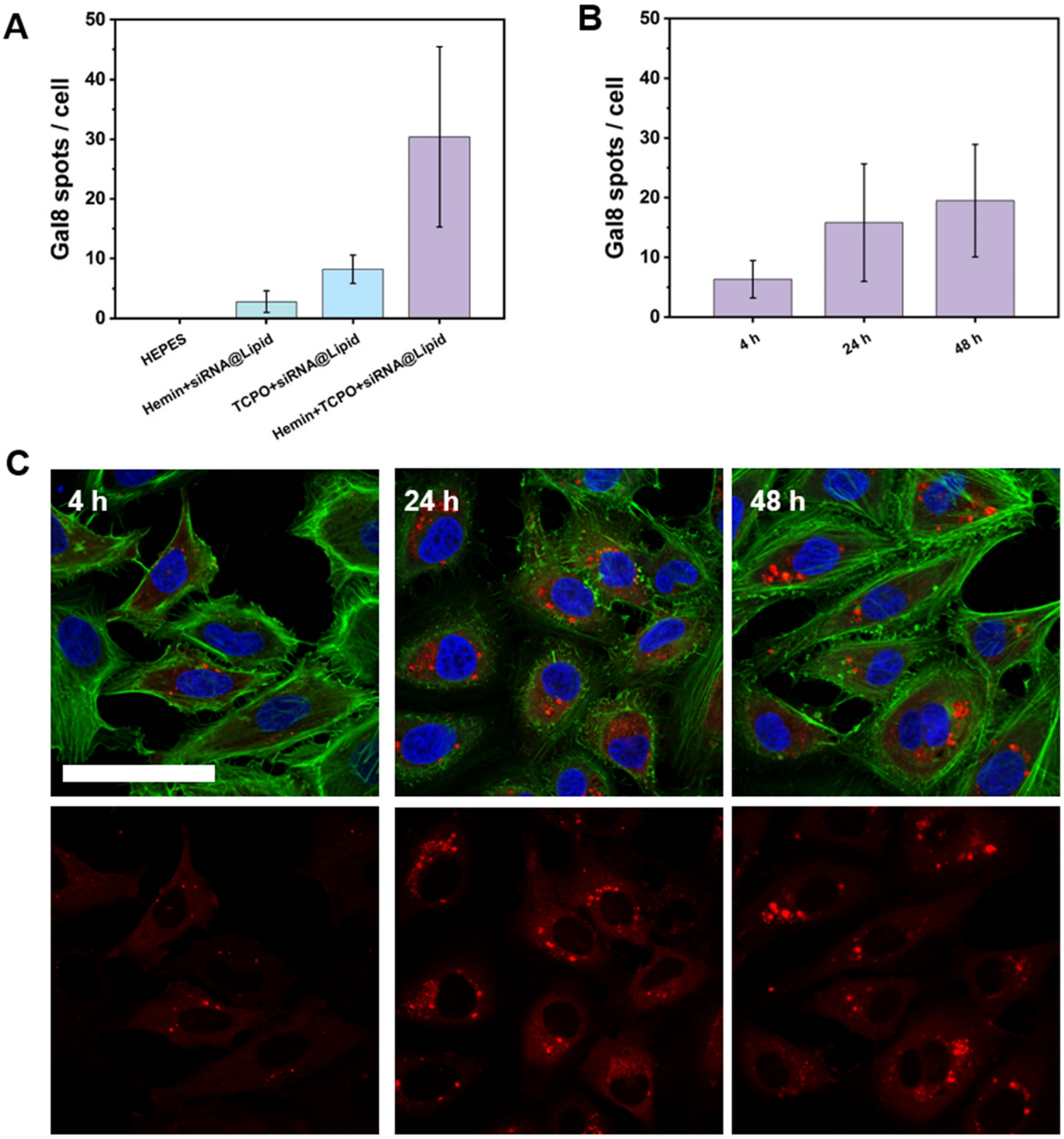

Figure S14. Endosomal escape assay. (A) Quantitative analysis of Gal8 spots per cell of cells treated with different formulations for 12 h. (B) Quantitative analysis and (C) confocal laser scanning microscopy images of Gal8 spots per cell of cells treated with Hemin+TCPO+siRNA@Lipid for 4 h, 24 h, and 48 h, respectively. siRNA, hemin, and TCPO concentrations were same with Figure 4C right side; the siRNA amount was 500 ng; scale bar, 100 μm.

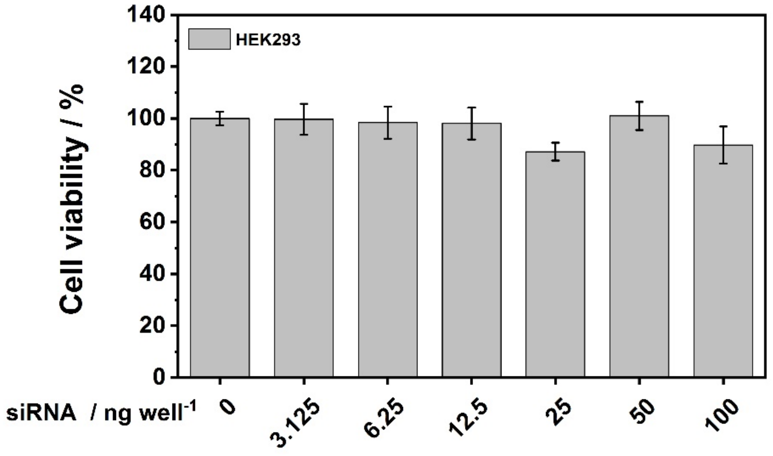

Figure S15. Cytocompatibility of Hemin+TCPO+siNrf2@Lipid in H_2O_2 low producing cells (HEK293). The TCPO amount was 8 μg well^{-1} and the hemin amount were 130 ng well^{-1}. Incubation time was 48 h.

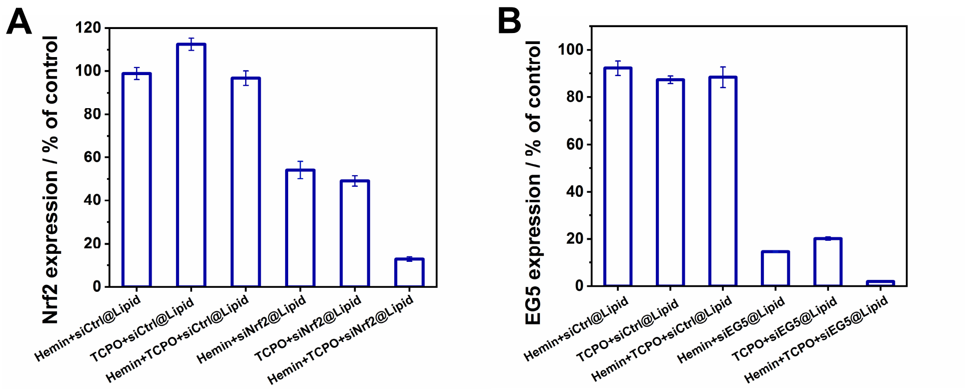

Figure S16. Analysis of gene silencing at mRNA expression level by reverse transcription-quantitative real-time PCR. mRNA expression in KB cells (120,000 cells/well) after (A) siNrf2-related LNPs and (B) siEG5-related LNPs incubation for 48 h. siRNA dose was 2 μg, TCPO dose was 400 μg, and hemin dose was 6.5 μg. For both cases, cells treated with Hemin+siCtrl@Lipid, TCPO+siCtrl@Lipid, and Hemin+TCPO+siCtrl@Lipid served as negative controls.

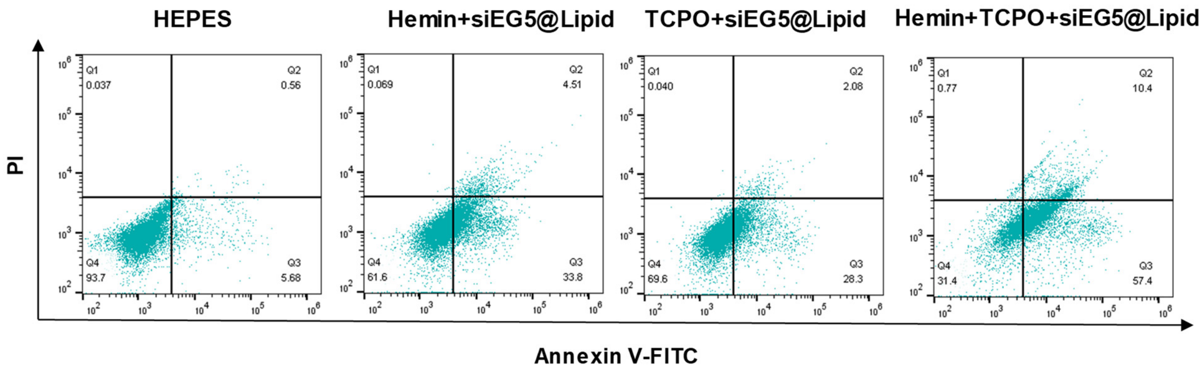

Figure S17. Cell apoptosis assay. Annexin V-fluorescein isothiocyanate/propidium iodide cell apoptosis analysis of KB cells after different treatments. The incubation time was 48 h.

Table S1. Molar ratio of each compound for LNPs with different N/P ratios.

	**N/P**	**Molar ratio of ionizable lipopeptide/cholesterol/DSPC/PEG-DMG**
Initial formulation	3.0 to 18	50/38.5/10/1.5 mol%
Optimized lipopeptide formulation	4.5	60/30.8/8/1.2 mol%
	9	75/19.25/5/0.75 mol%
	18	85.71/11/2.86/0.43 mol%
